# Physiologically Shrinking the Solution Space of a *Saccharomyces cerevisiae* Genome-Scale Model Suggests the Role of the Metabolic Network in Shaping Gene Expression Noise

**DOI:** 10.1371/journal.pone.0139590

**Published:** 2015-10-08

**Authors:** Baofang Chi, Shiheng Tao, Yanlin Liu

**Affiliations:** 1 College of Enology, Northwest A&F University, Yangling, Shaanxi, China; 2 Bioinformatics Center, Northwest A&F University, Yangling, Shaanxi, China; 3 College of Life Sciences and State Key Laboratory of Crop Stress Biology in Arid Areas, Northwest A&F University, Yangling, Shaanxi, China; University Paris South, FRANCE

## Abstract

Sampling the solution space of genome-scale models is generally conducted to determine the feasible region for metabolic flux distribution. Because the region for actual metabolic states resides only in a small fraction of the entire space, it is necessary to shrink the solution space to improve the predictive power of a model. A common strategy is to constrain models by integrating extra datasets such as high-throughput datasets and C^13^-labeled flux datasets. However, studies refining these approaches by performing a meta-analysis of massive experimental metabolic flux measurements, which are closely linked to cellular phenotypes, are limited. In the present study, experimentally identified metabolic flux data from 96 published reports were systematically reviewed. Several strong associations among metabolic flux phenotypes were observed. These phenotype-phenotype associations at the flux level were quantified and integrated into a *Saccharomyces cerevisiae* genome-scale model as extra physiological constraints. By sampling the shrunken solution space of the model, the metabolic flux fluctuation level, which is an intrinsic trait of metabolic reactions determined by the network, was estimated and utilized to explore its relationship to gene expression noise. Although no correlation was observed in all enzyme-coding genes, a relationship between metabolic flux fluctuation and expression noise of genes associated with enzyme-dosage sensitive reactions was detected, suggesting that the metabolic network plays a role in shaping gene expression noise. Such correlation was mainly attributed to the genes corresponding to non-essential reactions, rather than essential ones. This was at least partially, due to regulations underlying the flux phenotype-phenotype associations. Altogether, this study proposes a new approach in shrinking the solution space of a genome-scale model, of which sampling provides new insights into gene expression noise.

## Introduction

As a powerful tool for biological interpretation and discovery, over 100 genome-scale metabolic networks for more than 35 organisms have been reconstructed (see http://gcrg.ucsd.edu/InSilicoOrganisms/OtherOrganisms). These models have been widely used in molecular evolution studies [1–3], genome annotation [4, 5], metabolic engineering [6–9], and cellular phenotype predictions [10–12]. The broad applications of genome-scale metabolic network reconstructions largely owe its success to a constraint-based modeling strategy. Instead of developing a system of partial differential equations such as that observed in kinetic models, the constraint-based modeling method converts a metabolic network into a stoichiometric matrix [13]. This matrix ensures balance of metabolites flow throughout the network, thus resulting in hundreds of mass balance constraints. These, together with the upper and lower bounds of the metabolic reactions in the network, define a solution space where actual flux distributions reside [14]. However, in reality, the metabolic behavior of a cell is under complex physiological regulations, many of which have not been reflected by the constraints mentioned above. This implies that the region for biologically relevant flux distribution is represented by small fraction of the entire solution space of a constraint-based model (CBM). Therefore, shrinking the predicted solution space is necessary to improve the predictive ability of a CBM.

Diverse constraint-based reconstruction and analysis (COBRA) methods have been developed to probe the solution space [15]. These could be basically classified into two groups, namely, unbiased and biased methods. Unbiased methods, like the Monte Carlo Markov Chain sampling method, are mainly used to characterize all possible flux distributions within the solution space. However, cells do not utilize most of these flux distributions [16]. On the other hand, Flux Balance Analysis (FBA) is the most extensively employed in biased methods. By optimizing an objective function, FBA attempts to capture the region where real flux distributions are most likely to reside [17]. FBA is capable of performing quantitative predictions with relatively high accuracy. During the past decades, the explosive growth of omics data offered a good opportunity to reduce the solution space of a CBM by integrating experimental datasets, including gene expression data [12,18], proteomic data [19], and metabolite concentration data [20]. In particular, algorithm innovations related to transcriptomic data integration have undergone substantial progress in the past decades [21]. Recently, Herrgård’s group [22] systematically evaluated the predictive capabilities of eight classic methods. The results showed that in many conditions, these methods do not outperform simple FBA by using growth maximization and parsimony criteria when making phenotype predictions. Other studies have also described the limitations of the strategy [12, 23]. Therefore, alternative approaches in locating real flux distributions are warranted.

Extensive experimental evidences suggest that cellular phenotypes could be synergistically regulated by global biological regulatory factors under certain rules [24–27]. Compared to mRNA levels, protein abundance, and metabolite concentration in cells, metabolic flux levels are more capable of reflecting cellular phenotypes [28]. Therefore, phenotype-phenotype correlations are very likely to be observed at the metabolic flux level. Exploring metabolic flux phenotype correlations would benefit certain bounds refinements of reactions, further contributing to CBM solution space shrinkage. Experimentally identified metabolic flux data on *S*. *cerevisiae* has been rapidly accumulating in the past decades. It has laid a solid foundation on flux phenotype correlation analysis; however, this kind of data has not been extensively utilized. In the present study, a meta-analysis of experimental flux data from publications has been performed to shrink the solution space of the yeast CBM.

A refined model benefits biological discoveries. In the present study, we applied the refined solution space of a *S*. *cerevisiae* model to explore the influence of the metabolic network on gene expression noise. Gene expression noise is defined as the stochastic fluctuation of a gene’s expression at the protein level between isogenic cells under identical environment [29]. It is very important to understand the regulatory mechanism of gene expression noise because small variations at the noise level could result in a dramatic change at the phenotype level [30]. Previous studies have indicated that gene expression noise is regulated by several factors, including the structure of genes [31, 32], gene essentiality [31], gene expression level [30, 33], transcription/translation rate [34–36], chromatin remodeling [31,37], and regulation network [38–40]. A recent study indicated that gene expression noise could propagate and cause fluctuations in growth through corresponding metabolic reactions [41]. However, the interaction between gene expression noise and the metabolic network remains elusive.

In the present study, we first determined the correlations among several metabolic flux phenotypes through a systematic review of experimentally measured metabolic flux data. The correlations were quantified and imposed into a *S*. *cerevisiae* CBM to refine its solution space. Using the refined model, we examined whether the metabolic network influences expression noise of enzyme-coding genes.

## Results

### Correlated metabolic flux phenotype quantification

All the metabolic flux data we collected were measured under the conditions that the yeast strains were cultivated aerobically with glucose as the only carbon source (see **Materials and Methods**; **S1 Dataset**). Because sugar uptake rate plays a central role in shaping the global metabolic system under a carbon-limited condition [26], the relationship of other flux phenotypes to glucose uptake rates was explored. In an aerobic condition, *S*. *cerevisiae* can be mainly in any of two metabolic states, respiration and fermentation. In the former state, glucose is completely oxidized into CO_2_ through the mitochondrial respiration pathway, whereas in the latter, glucose is predominantly fermented into ethanol. Because of the existence of two metabolic patterns, it is necessary to identify the metabolic state that the *S*. *cerevisiae* is in prior to exploring flux-flux phenotype correlations.

**Fig 1:**
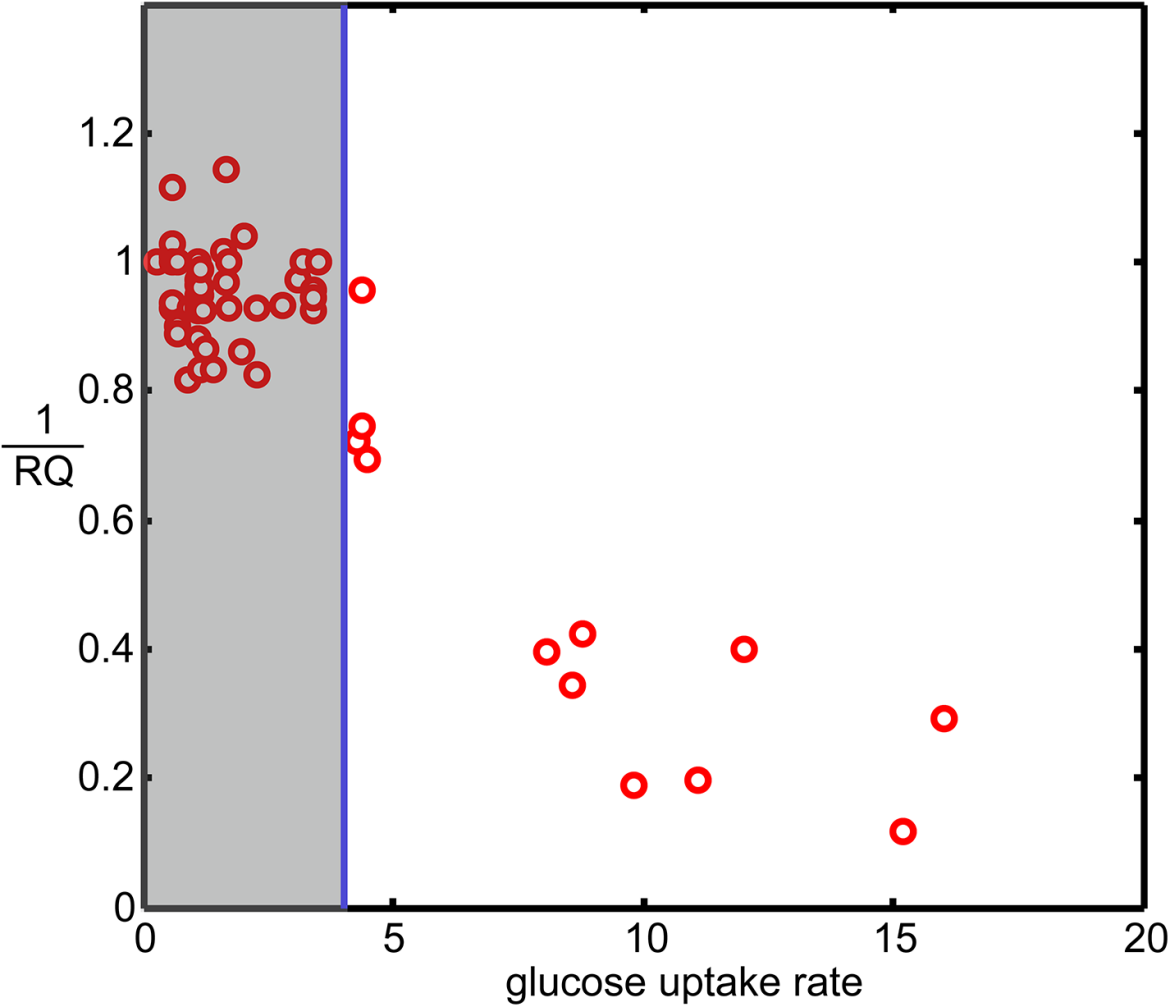
Distribution of RQ values at different glucose uptake rates ($\frac{1}{RQ}$ was used for the y-axis).

Because the respiratory quotient (RQ) is an indicator of metabolic states, RQ values against glucose uptake rates (For convenience, $\frac{1}{RQ}$ was used for the y-axes) were plotted. **Fig 1**shows that when the glucose uptake rate was <4mmol/ (g (DW)·h), the $\frac{1}{RQ}$ values fluctuate within the range of 0.81–1.06. This finding was suggestive of the dominant role of respiration over fermentation. When the glucose uptake rate was >4 mmol/ (g (DW)·h), the $\frac{1}{RQ}$ values gradually decreased as glucose uptake rate increased, which was indicative of a metabolic pattern transition from respiration to the fermentation state. This result is consistent with the study conducted by Postma*et al*. [42]. Therefore, the glucose uptake rate “4 mmol/ (g (DW)·h)” was defined as a “transition marker” for discriminating metabolic states.

**Fig 2:**
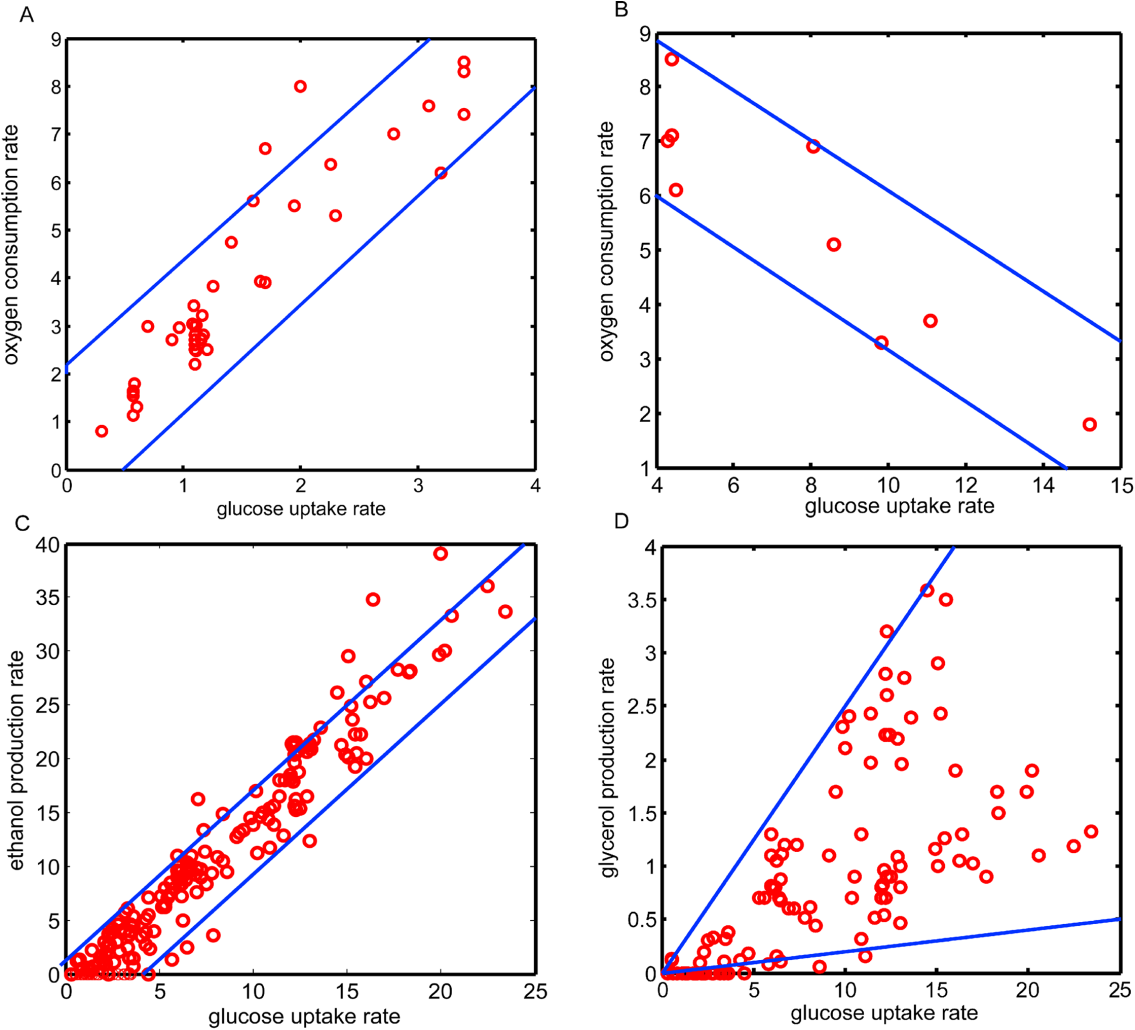
Metabolic flux phenotype-phenotype correlations. Red circles denote experimentally identified data. Blue lines indicate the upper (lower) bound based on regression analysis result. (**A**) O_2_ consumption rate versus glucose uptake rate (<4mmol/ (g (DW)·h)). (B) O_2_ consumption rate versus glucose uptake rate (>4mmol/ (g (DW)·h). (**C**) Ethanol production rate versus glucose uptake rate; (**D**) Glycerol secretion rate versus glucose uptake rate.

Next, O_2_ uptake rate, ethanol production rate, and glycerol secretion rate against glucose uptake rate were plotted, respectively. Results showed that when glucose uptake rate was <4 mmol/ (g (DW)·h), the O_2_ uptake rates positively correlated with glucose uptake rates (**Fig 2(A)**). On the other hand, when the glucose uptake rate was >4 mmol/ (g (DW)·h), the O_2_ uptake rate negatively correlated with the glucose uptake rate(**Fig 2(B)**). Unlike the O_2_uptake rate, ethanol production rates were always positively correlated with glucose uptake rates, independent of the metabolic state of yeast (**Fig 2(C)**). Glycerol secretion rates were correlated with glucose uptake rates (**Fig 2(D)**). Based on these findings, the correlation between the three flux phenotypes (O_2_ uptake rate, ethanol production rate, and glycerol secretion rate) and glucose uptake rate was quantified (**Table A in S3 File**).

### Prediction of the upper and lower bounds of the growth rate under aerobic conditions

**Fig 3:**
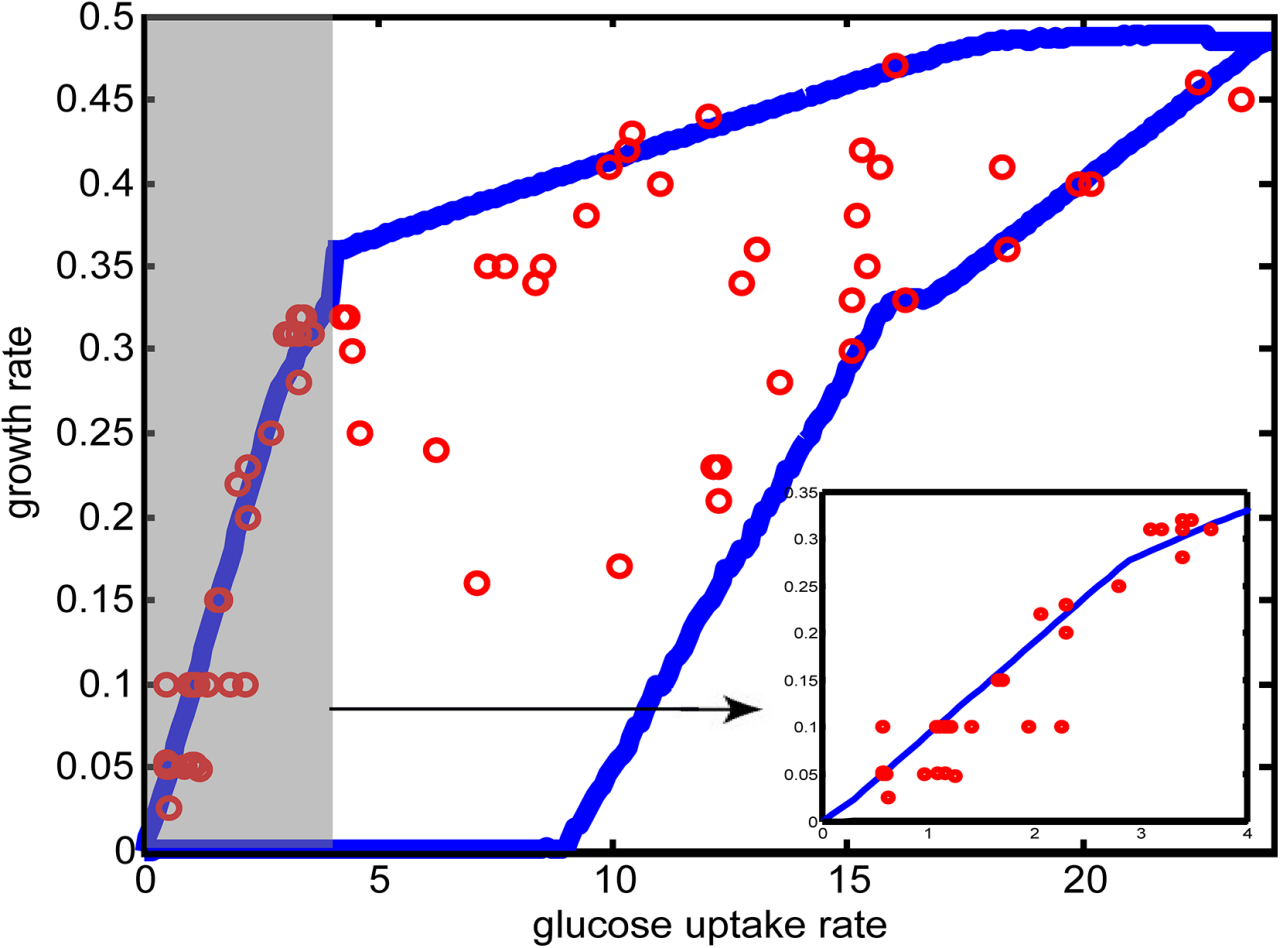
Comparison of predicted lower (upper) bound for growth rate and experimentally identified growth rates. The two blue lines indicate the predicted upper and lower bound for growth rate. The red points denote experimentally identified growth rates. **Inset**: When the glucose uptake rate was <4 mmol/(g(DW)·h), experimentally identified growth rates fitted well with the predicted upper bound for growth rate.

With the refined bounds for O_2_ uptake rate, ethanol production rate, and glycerol secretion rate, FBA was employed to predict the upper and lower bounds of growth rate (see **Materials and Methods**). **Fig 3**shows that almost all the experimentally identified growth rates were within the predicted space, indicating that our strategy was suitable for the refinement of flux bounds. In addition, when the glucose uptake rate was <4 mmol/(g(DW)·h), the experimental data fitted well with the predicted upper bound (R^2^ = 0.83), indicating the dominant role of the respiration pathway in supplying energy for growth. When the glucose uptake rate was >4mmol/ (g(DW)·h), the data points dispersed in the space. This finding suggested that once the nutrients in the environment are sufficient to support a high glucose uptake rate, *S*. *cerevisiae* would be relaxed from evolutionary pressures for survival. Under this condition, “biomass growth” is just one of the several goals that a cell strives to achieve [43], and growth rate becomes a yeast strain-dependent phenotype.

### Metabolic flux moderately correlates with mRNA level and protein abundance

Several studies have revealed a close relationship between metabolic flux and gene expression for various pathways in *S*. *cerevisiae* [24, 44, 45], but only a few studies directly identify this relationship [46]. In the present study, the average metabolic flux (AF) level of each metabolic reaction was estimated and compared to the corresponding gene expression level. **Table 1**showed that AF moderately correlated with mRNA level and protein abundance, which was consistent the findings of a previous study [46]. Contrary to the results of Bilu [46], our analysis suggested that the correlation between AF and protein abundance level is stronger than that between AF and mRNA level. This result reflected the fact that compared to mRNA level, protein abundance is more closely linked to metabolic flux level.

**Table 1:** Spearman rank correlation between metabolic flux level and mRNA level/protein abundance.

Data type	Correlation	p-value	Number of genes	Reference
mRNA number	0.26	9.13E^-05^	219	This study
mRNA number	0.37	1.00E^-12^	356	Bilu*et al*. 2006
mRNA number	0.35	2.00E^-11^	343	Bilu*et al*. 2006
Protein abundance	0.32	7.31E^-06^	186	This study
Protein abundance	0.22	4.00E^-04^	259	Bilu*et al*. 2006

### Metabolic flux fluctuation moderately correlates with gene expression noise

**Fig 4:**
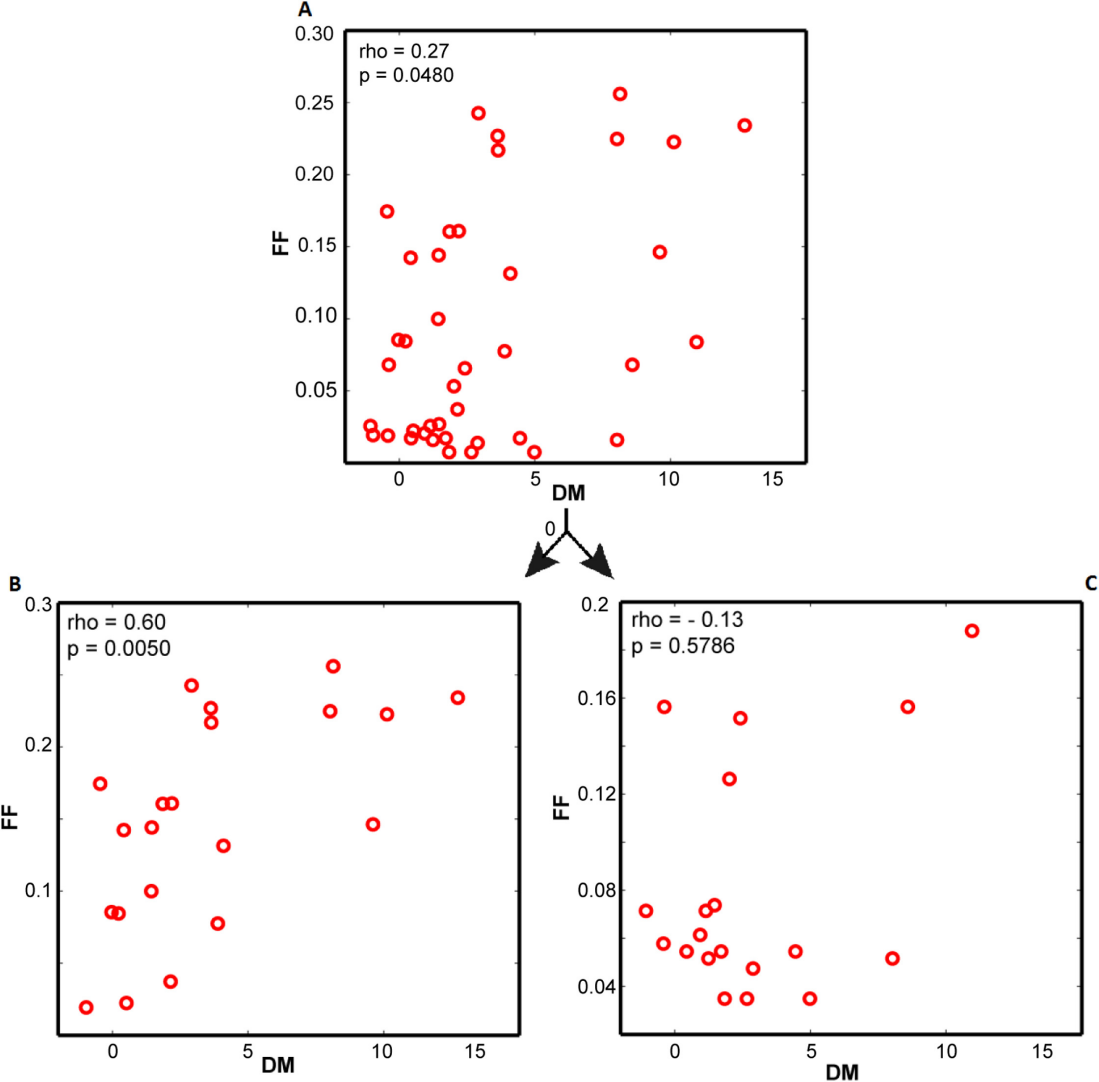
Correlations between flux fluctuation and gene expression noise. (**A**) Correlation between FF and DM for DSR genes. (**B**) Correlation between FF and DM for NER genes. (**C**) Correlation between FF and DM for ER genes.

Gene expression noise describes the cell-to-cell differences in protein level while maintaining homeostasis. In 2006, Newman *et al*. conducted a systematical research on gene expression noise of *S*. *cerevisiae*. They proposed DM (which is short for distance to median) value as a measure of gene-specific noise (see **Materials and Methods**). The noise is able to propagate and cause fluctuations in growth through corresponding metabolic reactions [41]. To know whether the metabolic network has influence on gene expression noise, we estimated metabolic flux fluctuation (FF) level of each reaction. FF could be regarded as reaction's intrinsic trait given by the metabolic network (see **Materials and Methods**), and was compared with corresponding DM value. Results showed no significant correlation between FF and DM in all enzyme-coding genes (data not shown). A possible reason is that for many reactions, the flux levels are not controlled by enzyme concentrations, but by ubiquitous allosteric regulatory mechanisms [23]. Thus, we next examined the genes that were mostly likely to be related to enzyme-dosage sensitive reactions (DSRs). According to Newman *et al*., protein abundance levels are measured in both rich (YEPD) and minimal media (SD) [24]. Between the two conditions, several genes were differentially expressed, whereas others remained stable. It is reasonable to assume that the reactions that were catalyzed by enzymes whose level were divergently expressed between the two conditions were more likely to be DSRs. Therefore, the ratio of protein abundance obtained from SD to those obtained from YEPD was used for DSR gene retrieval. Only the genes whose expression levels follow the expression,|Log_2_(SD/YEPD)| > 1,were considered as DSR genes. Finally, a total of 41 genes were obtained and subjected to correlation analysis. In the DSR genes, FF moderately correlated with DM (rho = 0.27, p = 0.0480; **Fig 4(A)**). To ensure the robustness of this result, three different cutoffs (log_2_(SD/YEPD)>0.5,0.7,1.0 respectively) were used to retrieve DSR genes for correlation calculations. The correlations between FF and DM were always positive(rho values ranging from 0.26 to 0.58; p<0.05). The more stringent the cutoff, the stronger the correlation. These findings ascertained that for DSR genes, the level of gene expression noise was subject to the tolerance of the metabolic network to flux fluctuations.

### Reaction essentiality is related to DM and FF coupling

Gene expression noise undergoes selection pressures when it is beneficial or detrimental to the survival of an organism [30–32]. However, that selection pressure for traits that are not vital to cell growth may be relaxed. To determine potential influences of reaction essentiality on FF and DM correlation, the 41 DSR genes were subdivided into “essential reaction” and “non-essential reaction” groups. Among the 41 genes, 20 were associated with essential reactions, whereas 21 were associated with non-essential reactions. Correlation analysis indicated that for the genes corresponding to non-essential reactions, FF strongly correlated with DM (rho = 0.60, p = 0.0050; **Fig 4(B)**). However, we did not observe a significant correlation in the genes that corresponded to essential reactions (**Fig 4(C)**).

**Fig 5:**
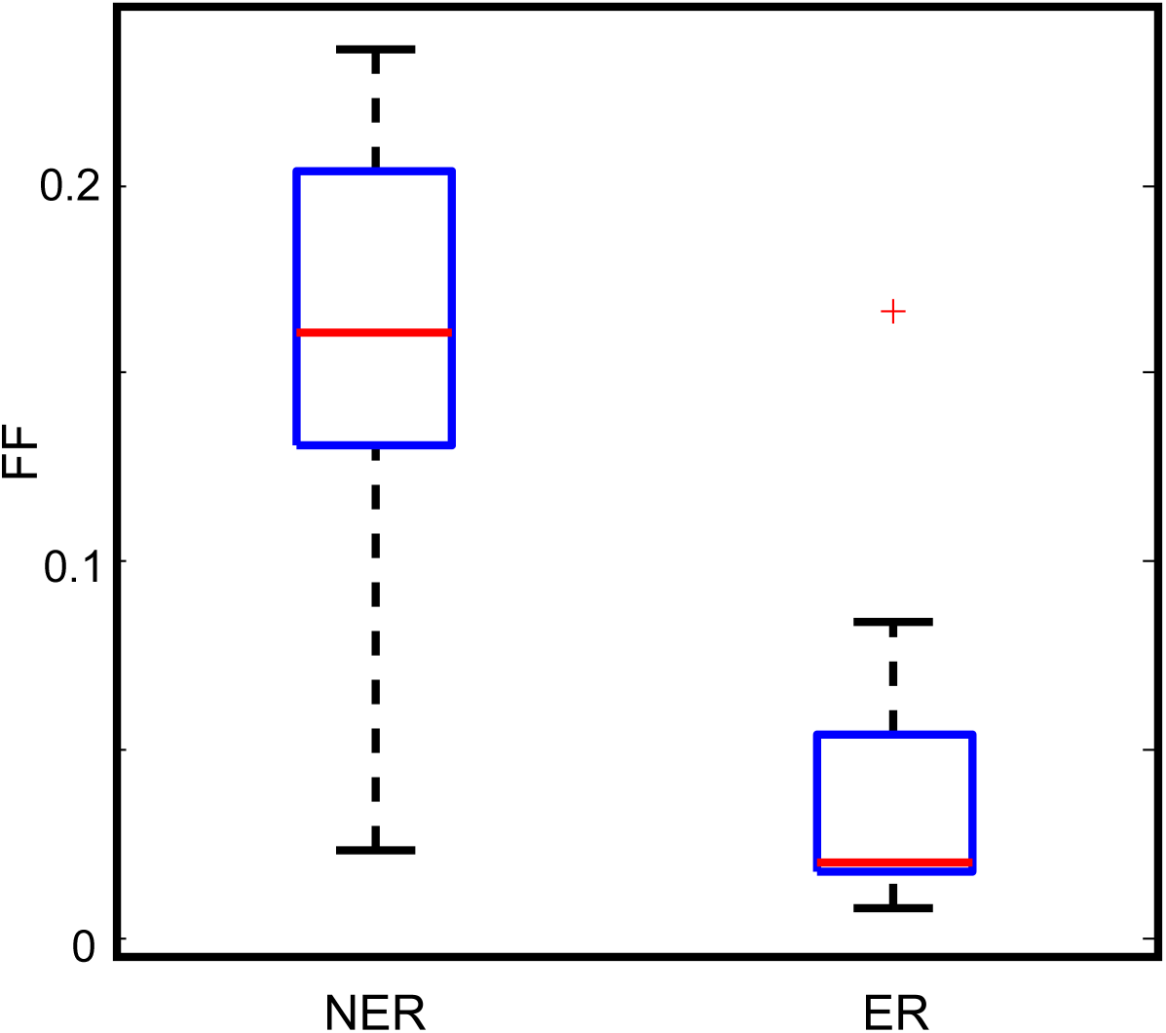
The FF values of the non-essential reaction (NER) group were generally higher than those of the essential reaction (ER) group.

**Fig 6:**
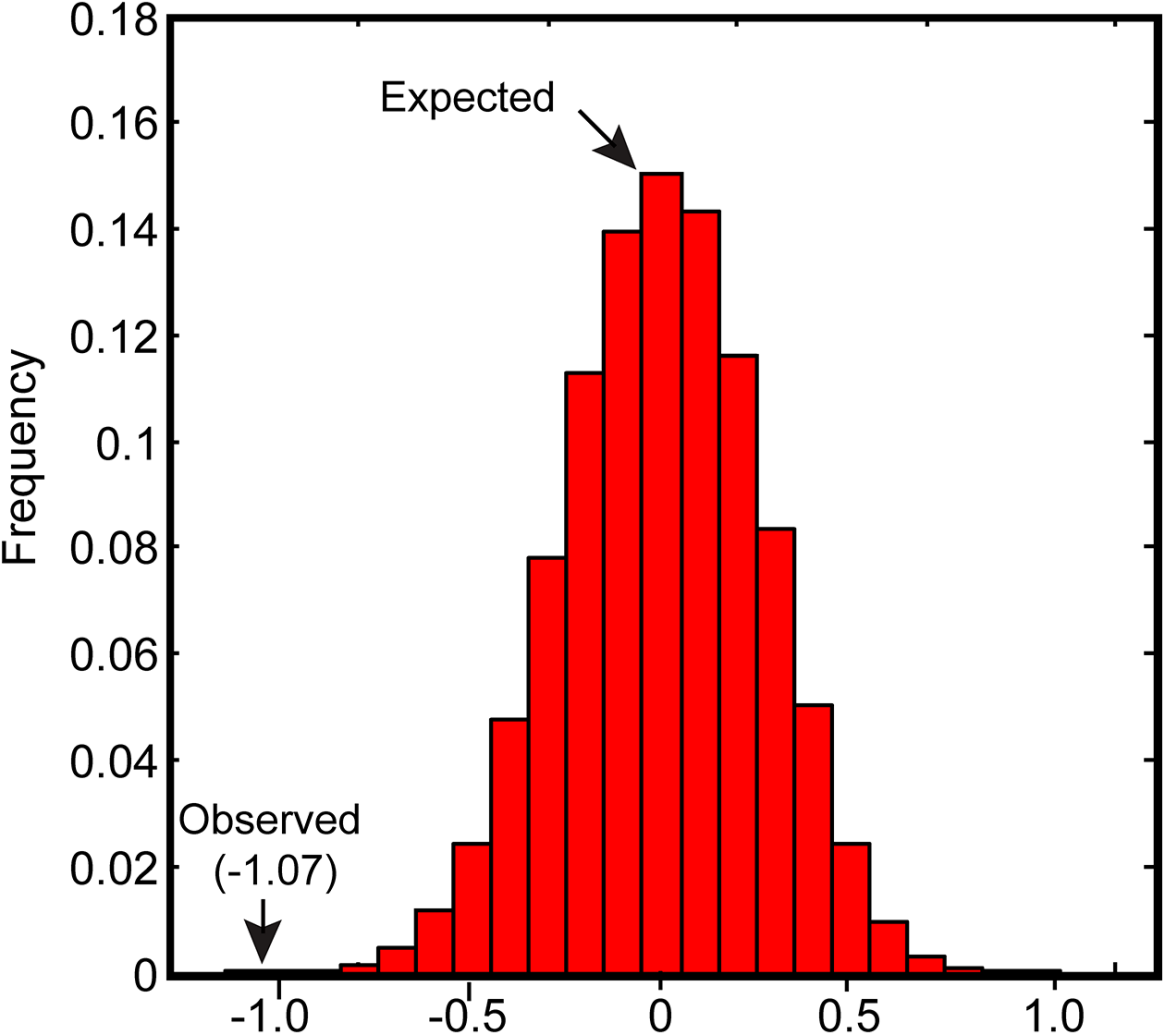
The observed differences in average FF values between the ER and NER groups are statistically significant. The x-axis shows the average FF difference between the ER group and the NER group in 10,000 random simulations. The y-axis indicates the number of simulated FF differences in a bin of width 0.1.

The DM and FF values between the essential and non-essential reaction groups were also compared. While no obvious differences were observed in the average DM values between the two groups, the FF values of non-essential reactions were generally higher than those of essential reactions (**Fig 5**). A simulation test was also performed to ensure the robustness of this result (see **Materials and Methods**), which showed that the simulated difference values between two groups were normally distributed around 0, with a standard deviation *σ* = 0.26. The observed difference value was at least four standard deviations away from the simulated mean difference. Therefore, the observation that FF values of non-essential reactions were higher than those of essential ones occurred by chance was highly unlikely (**Fig 6**).

To avoid the possibility that all the results obtained in this section were sampling artifacts of a specific part or the entire solution space, FF values were re-calculated by sampling another part of the shrunken space where the glucose uptake rate fluctuates within 20-22mmol/(g (DW)·h). These FF values were also used in the correlation analysis, and the results were the same as those obtained earlier (**Fig B in S3 File**).

### Physiological regulations mitigate FF and DM coupling

**Fig 7:**
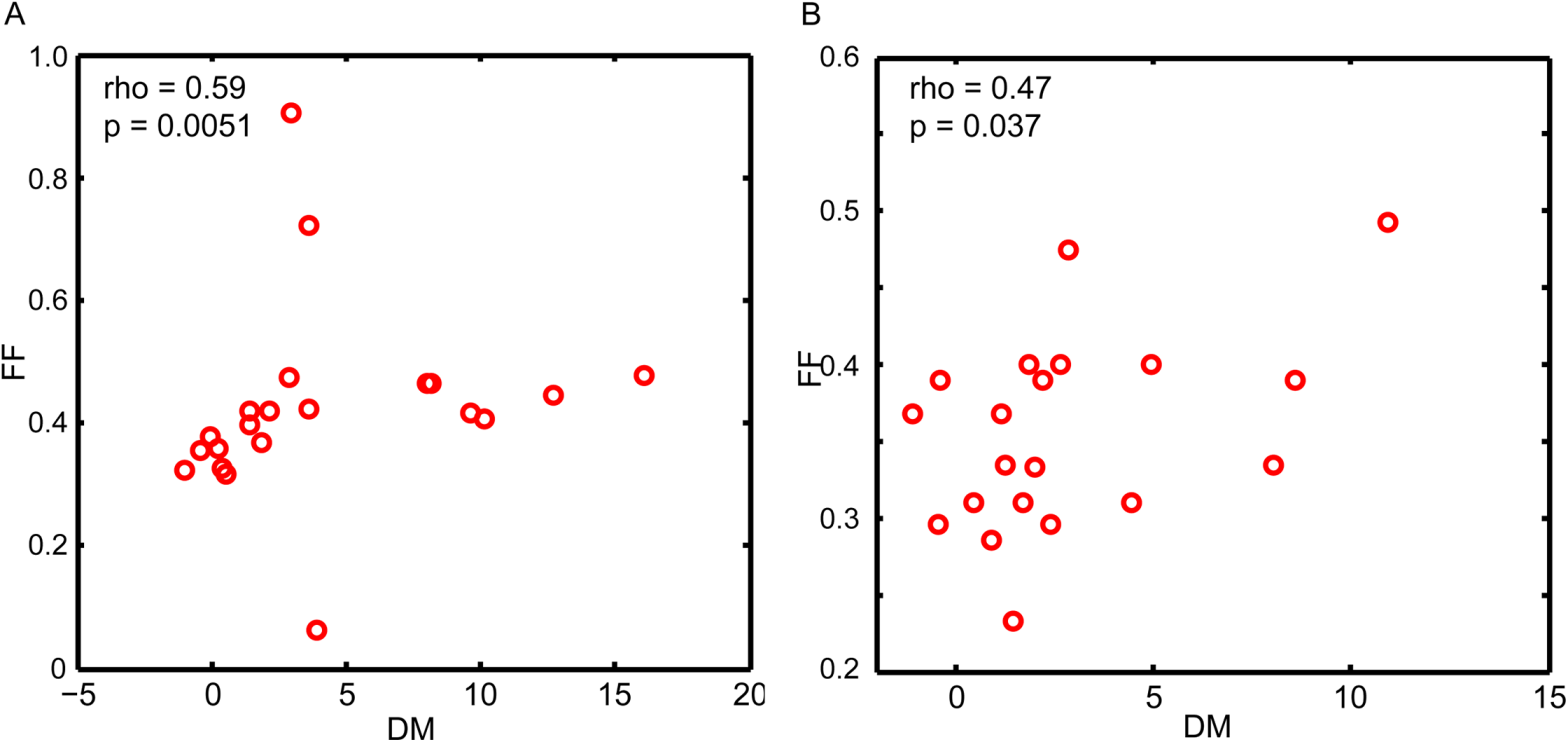
(A) Correlation between FF and DM in NER genes. (B) Correlation between FF and DM in ER genes. The FF values were calculated by sampling the entire yeast CBM solution space without the extra constraints.

In the previous section, we revealed the difference between “non-essential reaction”-associated and “essential reaction”-associated genes in FF and DM coupling. The difference was speculated to be related to the flux phenotype-phenotype correlations that were quantified and imposed into the yeast model as additional constraints. To test this hypothesis, the FF values of DSR genes were re-calculated by sampling the yeast model’s solution space without the constraints. Removal of the constraints did not remarkably affect the strong correlation with the “non-essential reaction”-related genes (rho = 0.59, p = 0.0051; **Fig 7(A)**). However, for genes related to essential reactions, newly calculated FF values strongly correlated with the DM values (rho = 0.47, p = 0.037; **Fig 7(B)**). These findings indicated that the global regulations underlying the phenotype-phenotype correlations mitigated the correlation between FF and DM in genes corresponding to essential reactions.

### Flux constraint strength is not involved in expression noise regulation

**Fig 8:**
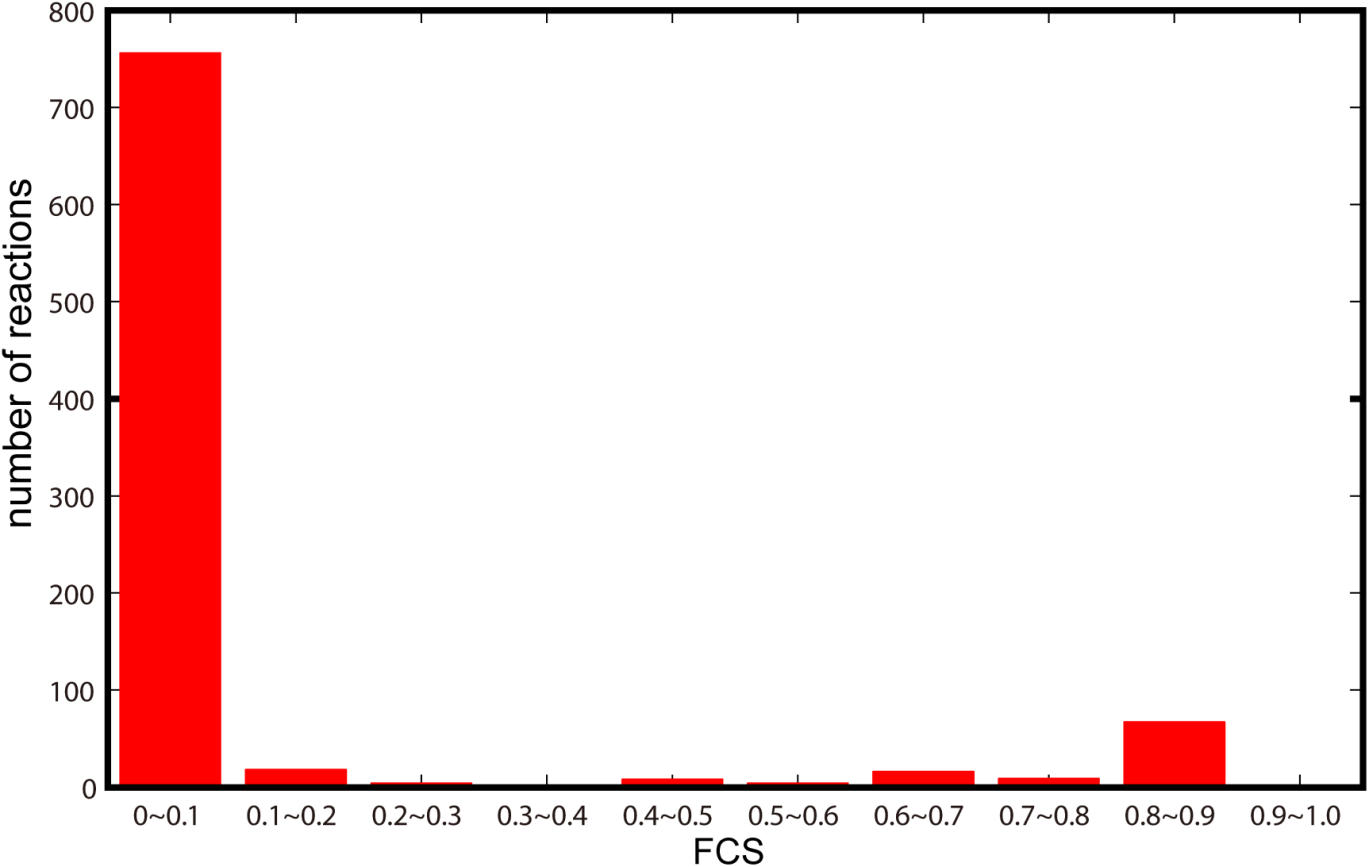
The number of genes under different constraint strengths. The smaller the FCS value, the greater the constraint strength.

In previous section, we have revealed that metabolic network has influence on gene expression noise. To know whether the influence is related to the extra constraint strength, we used FCS to estimate the level of flux constraints posed on each reaction (see **Materials and Methods**). **Fig 8**showed that the majority of FCS values range within 0–0.1 (746/885), indicating the broad effect of the regulatory mechanism underlying the constraints. However, we did not observed a significant correlation between flux constraint strength and gene expression noise (**Fig C in S3 File**). This implied that the regulatory strength does not play a key role in shaping gene expression noise.

## Discussion

Several studies have identified the limitations of using transcriptomic data in refining CBMs because complex regulations exist during transcription and translation. Because microbial metabolism is operated under certain global principles [24–27], we hypothesized that metabolic flux phenotypes are also under synergistical regulations, and thus correlate with each other. By summarizing the metabolic data that have accumulated in the past decades, we have identified several metabolic flux phenotype correlations, which were subsequently and successfully applied to the refinement of solution space. We expected to identify additional metabolic phenotype associations, for which the accumulated metabolic data during the past decades has served as solid foundation.

FF is an intrinsic trait of a metabolic reaction that is determined by the metabolic network where a reaction is located. In the present study, FF was found to correlate with DM for the DSR genes, suggesting that the metabolic network influenced gene expression noise. In particular, DSR genes corresponding to NERs showed a strong correlation between flux fluctuation and gene expression noise, which, however, was not observed in those related to essential reactions. This is, at least partially, due to the underlying flux phenotype-phenotype associations, because after the removal of the extra constraints, FF strongly correlated with DM for the DSR genes related to essential reactions. We thus concluded that the metabolic network is also an important determinant that constrains gene expression noise, especially for genes associated with the reactions that are not essential for organism's survival. However, for reactions that are vital to cell growth, stochastic fluctuations at the flux level led by gene expression noise might be detrimental. Under this condition, the cell adopts additional physiological regulatory mechanisms to precisely, rather than strongly, control gene expression, and thus reduce expression noise. By using this physiological response, the connections between the metabolic network and gene expression noise is mitigated.

In summary, the present study has revealed the associations among several metabolic flux phenotypes through *S*. *cerevisiae* physiological data mining. The integration of these flux phenotype-phenotype correlations into a yeast model has resulted in a substantial shrinkage of its solution space. By analyzing the biologically more relevant solution space, we concluded that the metabolic network contributes, sometimes predominantly, to shaping gene expression noise.

## Materials and Methods

### Data sources

#### Metabolic flux data

A comprehensive search of English-language published reports was performed using three search engines (PubMed, Google Scholar, and ScienceDirect). Several publisher archives, including “Applied and Environmental Microbiology”, “Yeast”, “Enzyme and Microbial Technology”, “Applied Microbiology Biotechnology”, and “Microbial Cell Factories”, were also thoroughly surveyed. The search terms included "*Saccharomyces cerevisiae*" or "*S*. *cerevisiae*" or "Yeast," plus “physiology” or "physiological," plus “metabolic” or “metabolism”. After removing duplicates, a total of 160 full-text articles were obtained. The content of these articles were reviewed and 96 of these were accepted (**S2 File**). The retrieved metabolic data met following criteria:

1. The unit of measurement for growth rate is “*h* ^−1^”; The units of measurement for other metabolic flux data is (or could be converted to)“mmol/ (g (DW)·h)”;
2. The yeast strains used in experiments did not undergo any artificial genetic manipulation at the molecular level such as gene deletion or overexpression;
3. All the wild-type yeast strains were cultivated aerobically in a steady state; and
4. Glucose was the only carbon source for yeast, and carbon was the only nutrient limitation of the culture.

After selection according to these criteria, we aggregated and normalized the data to generate a metabolic flux dataset (**S1 Dataset**), which includes glucose uptake rates, O_2_ uptake rates, CO_2_ production rates, respiratory quotient (RQ) values, ethanol production rates, and glycerol secretion rates.

#### Protein abundance

We considered three quantitative genome-wide measurements of protein abundance: genome-scale data from the study conducted by Ghaemmaghami *et al*. [47], and two genome-scale measurements in two conditions as performed by Newman *et al*. [29].

#### mRNA level

We considered three genome-wide measurements of mRNA levels according to Holstege *et al*. [48], Ingolia *et al*. [49], and Wang *et al*. [50].

#### Gene expression noise

We considered two yeast genome-wide datasets of gene expression noise level based on the report of Newman *et al*. [29]. In their work, DM was used as a measure of gene expression noise. DM was defined as the difference of the gene specific noise and the median noise for proteins with the same abundance. In this way, DM represents gene-specific noise independent of the corresponding gene expression level.

For protein abundance, mRNA levels, and gene expression noise, we averaged across data sets (after normalizing each data set by its mean) to minimize experimental noise.

### Quantification of metabolic flux phenotype-phenotype correlations and identification of flux boundaries

We examined the relationship between the glucose uptake phenotype and three metabolic flux phenotypes, namely,O_2_ uptake rate, ethanol production rate, and glycerol secretion rate, respectively.

Because O_2_ uptake rate and ethanol production rate linearly well correlate with glucose uptake rate (**Fig 2**), we learned linear models for them. To ensure robustness and avoid publication bias in the linear model, a procedure analogous to leave-one-out cross-validation was performed [51]: The data from each paper were regarded as elementary dataset. All these datasets were aggregated to construct a “reference dataset”. Next, leave-one-elementary dataset-out (LOE) datasets (LDs) were constructed by iteratively omitting each elementary dataset from the reference dataset (If ***m*** elementary datasets are available for analysis, then ***m*** LOE datasets will be obtained). Linear regression analysis was conducted using the data from each LOE dataset, and ***m*** slope values and corresponding R^2^ values were obtained. The results showed that the ***m*** slope values were almost equal to each other, with R^2^>0.8, indicating the robustness of our modeling strategy.

By performing linear regression analysis, the slope value *α* (*α*_*oxy*_ for oxygen uptake versus glucose uptake curve; and *α*_*eth*_ for ethanol production versus glucose uptake curve) was defined. While fixing the *α* value, two *β* values, namely, *β*_*l*_ and *β*_*u*_, were set through visual inspection to define a space covering > 90% of the following experimental identified points:

$$lb_{oxy}=\alpha_{oxy}v_{glu}+\beta_{l\_oxy}$$

$$ub_{oxy}=\alpha_{oxy}v_{glu}+\beta_{u\_oxy}$$

$$lb_{eth}=\alpha_{eth}v_{glu}+\beta_{l\_eth}$$

$$ub_{eth}=\alpha_{eth}v_{glu}+\beta_{u\_eth}$$

where *v*_*glu*_ represents glucose uptake rate; and *lb* (*ub*) represents lower (upper) bound for ethanol (or O_2_) exchange rate.

Glycerol secretion rate did not correlate well with glucose uptake rate. Therefore, its bounds were defined, somewhat arbitrarily, by modifying the *α* value as follows:

$$lb_{gly}=\alpha_{gly}v_{glu}$$

$$ub_{gly}=\alpha_{gly}v_{glu}$$

where *lb*_*gly*_ (*ub*_*gly*_)represents the lower (upper) bound of the glycerol secretion rate.

All aforementioned *α* and *β* values are listed in **Table A in S3 File**. The newly refined glucose uptake-dependent bounds for O_2_ consumption rate, ethanol production, rate, and glycerol secretion rate are as follows:

$$lb_{oxy}\leq v_{oxy}\leq ub_{oxy};$$ (1)

$$lb_{eth}\leq v_{eth}\leq ub_{eth};\;\text{and}$$ (2)

$$lb_{gly}\leq v_{gly}\leq ub_{gly}.$$ (3)

### Prediction of the upper and lower bound for growth rate

*S*. *cerevisiae* genome-scale metabolic network model **iMM904** was used because of its high predictive power among available yeast models [52, 53]. To simulate yeast cultivated in glucose-limited media, fructose and ethanol uptake rates were fixed at 0 because these are potential carbon sources [52]. At each glucose uptake rate, *v*_*oxy*_, *v*_*eth*_ and *v*_*gly*_ values were uniformly sampled within the bounds defined by (1), (2), and (3), respectively, and the maximum growth *v*_*gro*_ rate that the yeast could achieve was calculated through FBA as follows:

$$\text{maximize}\;v_{gro}$$ (4)

subject to: **S•v** = 0

$$lb_{i}\leq v_{i}\leq ub_{i}\;v_{i}\in v$$

where *v*_*gro*_ indicates the flux of “biomass growth”;**S** is an M **×** N stoichiometric matrix (M is the number of metabolites, and N is the number of reactions);**v** is a vector containing metabolic flux *v*_*i*_ of N reactions; and *lb*_*i*_ (*ub*_*i*_) represents the lower (upper) bound for *v*_*i*_. When *lb*_*oxy*_ <0.016 mmol/ (g (DW)·h) at a certain glucose uptake rate, we set *lb*_*oxy*_ to 0.016 mmol/ (g (DW)·h) [5].

After repeating this process 10,000 times, the biggest (smallest) value in the 10,000 simulated growth rate values was regarded as the maximum (minimum) growth rate *v*_max_ (*v*_min_) the yeast was able to achieve under a glucose uptake rate. The *v*_max_ (*v*_min_) under glucose uptake rates ranging from 0 to 24 (step size = 0.1) were all computed.

Because the upper (lower) bound for growth rate was likely to be a combination of several linear functions (**Fig 3**), the predicted *v*_max_ (*v*_min_) was divided into several segments according to glucose uptake rates, for each of which linear regressions of the data were performed separately (**Fig A in S3 File**).The resulting equations were used as upper (lower) bound for the growth rate (All the equations are listed in **Table B in S3 File**). For simplicity, the bounds were presented as follows:

$$lb_{gro}\leq v_{gro}\leq ub_{gro}\;.$$ (5)

### Estimation of the average flux, flux fluctuation and flux constraint strength

The average flux level (AF), flux fluctuation (FF), and flux constraint strength (FCS) of each metabolic reaction were estimated by sampling *S*. *cerevisiae*'s solution space. The sampling was restricted to the part of solution space where glucose uptake rate fluctuates within 18-20mmol/ (g (DW)·h) for two considerations: (1) Fluctuations of catabolically active enzymes could lead to growth fluctuations [41]; and (2) Data on mRNA levels to which AF would be compared, and data for gene expression noise to which FF would be compared, were all collected from rich media, under which glucose uptake rate ranges within 0.36–0.40 [54]. The upper (lower) bound for *v*_*oxy*_, *v*_*eth*_ and *v*_*gly*_ were set according to (1), (2), and (3), respectively. Facilitated with these additional constraints, The shrunken solution space was fully sampled to generate 500,000 eligible flux distributions.

The sampled flux values of each metabolic reaction were extracted from the 500,000 flux distributions. Given a reaction, the top and bottom 5% simulated flux values were discarded. The remaining sampled flux values were used to calculate AF and FF as follows:

$$AF_{i}=\left|\frac{1}{2}\left(v_{i,\text{max}}+v_{i,\text{min}}\right)\right|$$

$$FF_{i}=\left|\frac{v_{i,\text{max}}-v_{i,\text{min}}}{\frac{1}{2}\left(v_{i,\text{max}}+v_{i,\text{min}}\right)}\right|$$

where *AF*_*i*_ is defined as average flux level of the *i*-threaction; and *FF*_*i*_ is the flux fluctuation of the *i*-th reaction. *v*_*i*,max_ (*v*_*i*,min_)represents the maximum (minimum) flux value among the sampled flux values of the *i*-th metabolic reaction.

To estimate the constraint strength posed on each reaction, we also sampled yeast model's solution space 500,000 times before imposing the extra constraints. After removing the top (bottom) 5% flux values of each reaction, FCS was defined as follows:

$$FCS_{i}=\left|\frac{v_{i,\text{max}}-v_{i,\text{min}}}{\left(v_{i,\text{max},ori}-v_{i,\text{min},ori}\right)}\right|$$

where *v*_*i*,max,*ori*_ (*v*_*i*,min,*ori*_) represents the *i*-th maximum (minimum) flux value, which was generated from the original solution space. Reactions and metabolites participating in the intracellular loops were not included in the analysis [45]. To exclude potential biases, reactions catalyzed by isozymes were also excluded in the correlation analysis, thus ensuring a *one gene-one reaction* relationship. Bi-directional reactions were also not involved in AF and FF calculation.

### Identification of essential and non-essential reactions

Setting “biomass growth” as the objective function to be optimized, FBA was conducted to predict the growth rate *v*_*ori*_ of yeast model iMM904 without any extra constraints. We iteratively set reaction flux *v*_*i*_ in the yeast model to 0 and employed FBA again to predict the growth rate *v*_*gro*,*i*_. When *v*_*gro*,*i*_ = 0, this *i*-th reaction was defined as an “essential reaction.” When *v*_*gro*,*i*_ = *v*_*ori*_, this *i*-th reaction was defined as a “non-essential reaction.”

### Simulation test for FF value differences

FF values of the 41 DSR genes were shuffled and randomly divided into two groups, one consisting of 20 values and the remaining values were binned into another one. Difference in average FF values was calculated by subtracting the average FF value in one group by that of another. This process was repeated 100,000 times.

### Statistical analysis

All simulations and statistical analyses were performed in **MATLAB**, which was interfaced with the **COBRA** toolbox and ‘*glpk*’ solver. All correlations described in this report are Spearman’s rank correlations.

## Supporting Information

S1 Dataset Normalized metabolic flux data used for analysis. (XLSX)

S1 File Flowchart of the selection process. (PDF)

S2 File The publications used for *S*. *cerevisiae* metabolic data extraction. (PDF)

S3 File Supplementary figures and tables. Regression analysis of growth rate against glucose uptake rate in each segment **(Figure A).**Correlations between metabolic flux and gene expression noise **(Figure B).**Relationship between metabolic flux constraint (FSC) and gene expression noise (DM) **(Figure C).**The newly defined bounds **(Table A).**Regression analysis of growth rate versus glucose uptake rate in each segments. (y **=** αx +β; y is for growth rate and x is for glucose uptake rate(**Table B**).
(PDF)
