# Supplementary figures and images for: Physiologically Shrinking the Solution Space of a Saccharomyces cerevisiae Genome-Scale Model Suggests the Role of the Metabolic Network in Shaping Gene Expression Noise
Source: PLoS One. 2015 Oct 8;10(10):e0139590. doi: 10.1371/journal.pone.0139590 (PMC4598104; doi:10.1371/journal.pone.0139590)

**S1 File Flowchart of the selection process**

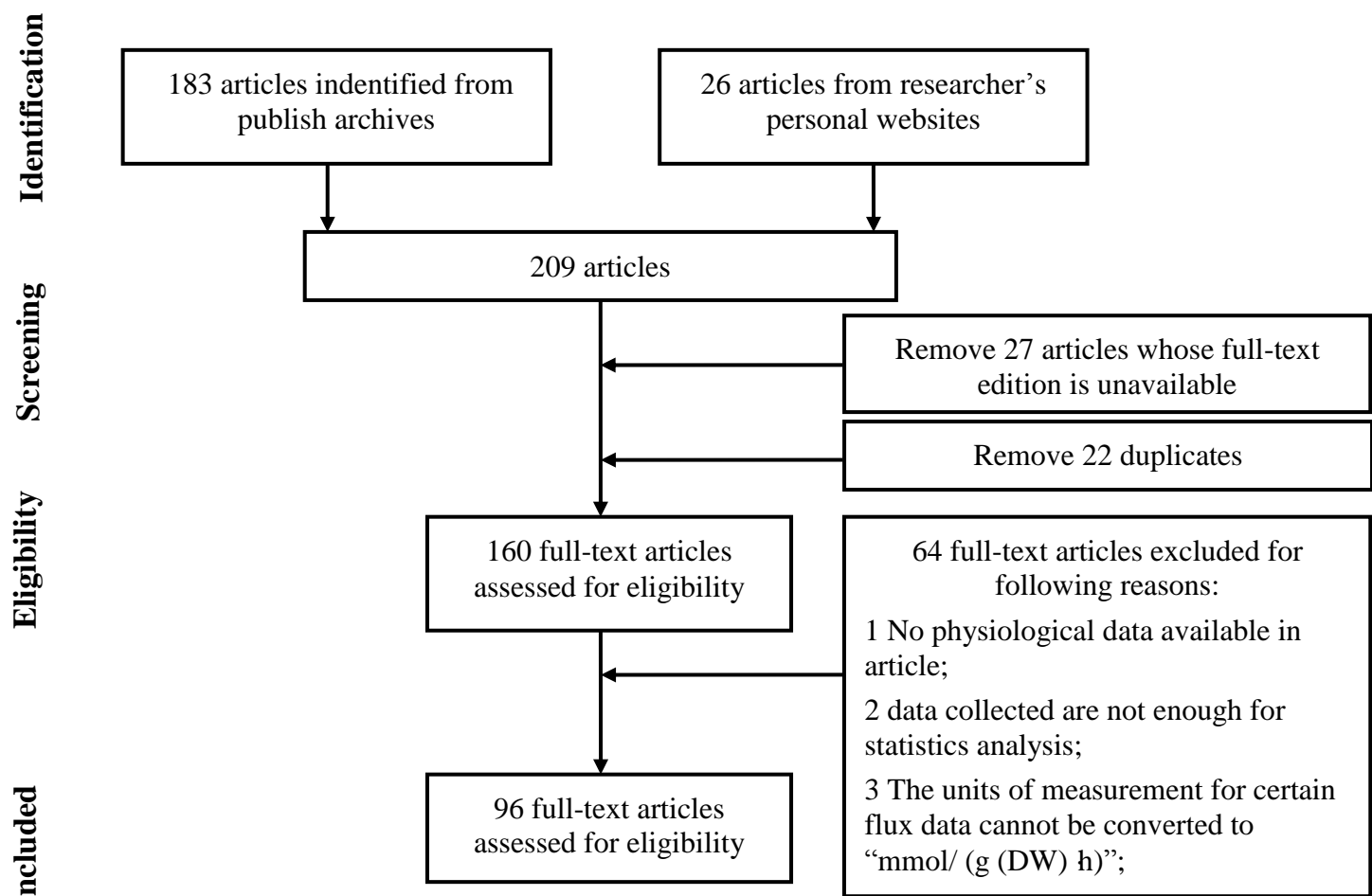

Supplement: S1 File — (PDF) [file pone.0139590.s002.pdf]
