# Supplementary material for: Physiologically Shrinking the Solution Space of a Saccharomyces cerevisiae Genome-Scale Model Suggests the Role of the Metabolic Network in Shaping Gene Expression Noise
Source: PLoS One. 2015 Oct 8;10(10):e0139590. doi: 10.1371/journal.pone.0139590 (PMC4598104; doi:10.1371/journal.pone.0139590)
Supplement: S2 File — (PDF) [file pone.0139590.s003.pdf]

Text S2. The publications used for S. cerevisiae metabolic data extraction

[1] Nissen TL, Hamann CW, Kielland-Brandt MC, Nielsen J, Villadsen J (2000) Anaerobic and aerobic batch cultivations of Saccharomyces cerevisiae mutants impaired in glycerol synthesis. *Yeast* 16: 463-474.
[2] Flikweert MT, Kuyper M, van Maris AJ, Kötter P, van Dijken JP, et al. (1999) Steady-state and transient-state analysis of growth and metabolite production in a *Saccharomyces cerevisiae* strain with reduced pyruvate-decarboxylase activity. Biotechnology and Bioengineering 66: 42-50.
[3] Vicente A, Castrillo JI, Teixeira JA, Ugalde U (1997) On-line estimation of biomass through pH control analysis in aerobic yeast fermentation systems. *Biotechnology and Bioengineering* 58: 445-450.
[4] van Hoek P, van Dijken JP, Pronk JT (2000) Regulation of fermentative capacity and levels of glycolytic enzymes in chemostat cultures of *Saccharomyces cerevisiae*. *Enzyme and Microbial Technology* 26: 724-736.
[5] Ciani M, Ferraro L, Fatichenti F (2000) Influence of glycerol production on the aerobic and anaerobic growth of the wine yeast Candida stellata. *Enzyme and Microbial Technology* 27: 698-703.
[6] Toivari MH, Aristidou A, Ruohonen L, Penttilä M (2001) Conversion of xylose to ethanol by recombinant *Saccharomyces cerevisiae*: importance of xylulokinase (XKS1) and oxygen availability. Metabolic Engineering 3: 236-249.
[7] Nissen TL, Kielland-Brandt MC, Nielsen J, Villadsen J (2000) Optimization of Ethanol Production in *Saccharomyces cerevisiae* by metabolic engineering of the ammonium assimilation. Metabolic Engineering 2: 69-77.
[8] Blom J, De Mattos MJ, Grivell LA (2000) Redirection of the respiro-fermentative flux distribution in Saccharomyces cerevisiae by overexpression of the transcription factor Hap4p. *Applied and Environmental Microbiology* 66: 1970-1973.
[9] Eliasson A, Christensson, Wahlbom CF, Hahn-Hägerdal B (2000) Anaerobic xylose fermentation by recombinant Saccharomyces cerevisiae carrying XYL1, XYL2, and XKS1 in mineral medium chemostat cultures. *Applied and Environmental Microbiology* 66: 3381-3386.
[10] Guo ZP, Zhang L, Ding ZY, Gu ZH, Shi GY (2011) Development of an industrial ethanol-producing yeast strain for efficient utilization of cellobiose. *Enzyme and Microbial Technology* 49: 105-112.
[11] Purwadi R, Taherzadeh MJ (2008) The performance of serial bioreactors in rapid continuous production of ethanol from dilute-acid hydrolyzates using immobilized cells. *Bioresource Technology* 99: 2226-2233
[12] Bro C, Regenberg B, Förster J, Nielsen J (2006) In silico aided metabolic engineering of *Saccharomyces cerevisiae* for improved bioethanol production. Metabolic Engineering 8: 102-111.
[13] Van Vleet JH, Jeffries TW, Olsson L (2008) Deleting thepara-nitrophenyl phosphatase (pNPPase), PHO13, in recombinant *Saccharomyces cerevisiae* improves growth and ethanol production on D-xylose. *Metabolic Engineering* 10: 360-369.
[14] Guo ZP, Zhang L, Ding ZY, Shi GY (2011) Minimization of glycerol synthesis in industrial ethanol yeast without influencing its fermentation performance. Metabolic Engineering 13: 49-59.
[15] Roca C, Haack MB, Olsson L (2003) Engineering of carbon catabolite repression in recombinant xylose fermenting *Saccharomyces cerevisiae. Applied Microbiology Biotechnology* 63: 578-583 doi: 10.1007/s00253-003-1408-2.
[16] Blank LM, Kuepfer L, Sauer U (2005) Large-scale ^{13}C-flux analysis reveals mechanistic principles of metabolic network robustness to null mutations in yeast. *Genome Biology* 6: R49 doi:10.1186/gb-2005-6-6-r49.

[17] Thomsson E, Larsson C, Albers E, Nilsson A, Franzén CJ, et al. (2003) Carbon starvation can induce energy deprivation and loss of fermentative capacity in *Saccharomyces cerevisiae*. *Applied and Environmental Microbiology* 69: 3251-3257.
[18] Schneider k, Krömer JO, Wittmann C, Alves-Rodrigues I, Meyerhans A, et al. (2009) Metabolite profiling studies in *Saccharomyces cerevisiae*: an assisting tool to prioritize host targets for antiviral drug screening. *Microbial Cell Factories* 8: 12 doi:10.1186/1475-2859-8-12.
[19] Lei F, Olsson L, Jørgensen SB (2003) Experimental investigations of multiple steady states in aerobic continuous cultivations of *Saccharomyces cerevisiae*. *Biotechnology and Bioengineering* 82: 766-777.
[20] Franzén CJ (2003) Metabolic flux analysis of RQ-controlled micro aerobic ethanol production by *Saccharomyces cerevisiae*. *Yeast* 220: 117-132.
[21] Nissen TL, Schulze U, Nielsen J, Villadsen J (1997) Flux distributions in anaerobic, glucose-limited continuous cultures of *Saccharomyces cerevisiae. Microbiology* 143: 203-218.
[22] Parachin NS, Bergdahl B, van Niel EWJ, Gorwa-Grauslund MF (2011) Kinetic modeling reveals current limitations in the production of ethanol from xylose by recombinant *Saccharomyces cerevisiae. Metabolic Engineering* 13: 508-517.
[23] Krahulec S, Petschacher B, Wallner M, Longus K, Klimacek M, et al. (2010) Fermentation of mixed glucose-xylose substrates by engineered strains of *Saccharomyces cerevisiae*: role of the coenzyme specificity of xylose reductase, and effect of glucose on xylose utilization. *Microbial Cell Factories* 9:16.
[24] Rossi G, Sauer M, Porro D, Branduardi P (2010) Effect of HXT1 and HXT7 hexose transporter overexpression on wild-type and lactic acid producing *Saccharomyces cerevisiae* cells. *Microbial Cell Factories* 9: 15.
[25] Larsson C, Nilson A, Blomberg A, Gustafsson L (1997) Glycolytic flux is conditionally correlated with ATP concentration in *Saccharomyces cerevisiae*: a chemostat study under Carbon-or Nitrogen-limiting conditions. *Journal of Bacteriology* 179:7243–7250.
[27] Vemuri GN, Eiteman MA, McEwen JE, Olsson L, Nielsen J (2007) Increasing NADH oxidation reduces overflow metabolism in *Saccharomyces cerevisiae*. *Proceedings of the National Academy of Sciences* 104: 2402-2407 doi: 10.1073/pnas.0607469104
[28] van Hoek P, van Dijken JP, Pronk JT (1998) Effect of specific growth rate on fermentative capacity of baker's yeast. *Applied and Environmental Microbiology* 64: 4226-4233.
[29] Cortassa S, Aon MA (1998) The onset of fermentative metabolism in continuous cultures depends on the catabolite repression properties of *Saccharomyces cerevisiae. Enzyme and Microbial Technology* 22:705-712.
[30] Heyland J, Fu J, Blank LM (2009) Correlation between TCA cycle flux and glucose uptake rate during respiro-fermentative growth of *Saccharomyces cerevisiae. Microbiology* 155: 3827-3837.
[31] Jouhten P, Rintala E, Huuskonen A, Tamminen A, Toivari M, Wiebe M, Ruohonen L, Penttilä M, Maaheimo H (2008) Oxygen dependence of metabolic fluxes and energy generation of *Saccharomyces cerevisiae* CEN.PK113-1A. *BMC Systems Biology* 2:60 doi:10.1186/1752-0509-2-60.
[32] Wisselink HW, Cipollina C, Oud B, Crimi B, Heijnen JJ, Pronk JT, van Maris AJA (2010) Metabolome, transcriptome and metabolic flux analysis of arabinose fermentation by engineered *Saccharomyces cerevisiae. Metabolic Engineering* 12: 537-551.
[34] Ostergaard S, Olsson L, Johnston M, Nielsen J (2000) Increasing galactose consumption by *Saccharomyces cerevisiae* through metabolic engineering of the GAL gene regulatory network. *Nature Biotechnology* 18: 1283-1286.
[35] Diderich JA, Schepper M, van Hoek P, Luttik MAH, van Dijken JP et al. (1999) Glucose uptake kinetics and transcription of HXT Genes in chemostat cultures of *Saccharomyces cerevisiae. Journal of Biological Chemistry* 274:15350-15359. doi:10.1074/jbc.274.22.15350.

[36] Postmus J, Tuzun I, Bekker M, Müller MH, de Mattos MJT, et al (2011) Dynamic regulation of mitochondrial respiratory chain efficiency in *Saccharomyces cerevisiae. Microbiology* 157: 3500-3511.
[37] Raab AM, Hlavacek V, Bolotina N, Lang C (2011) Shifting the fermentative/oxidative balance in *Saccharomyces cerevisiae* by transcriptional deregulation of Snf1 via over-expression of the upstream activating kinase Sak1p. *Applied and Environmental Microbiology* 77: 1981-1989.
[38] Otterstedt K, Larsson C, Bill RM, Ståhlberg A, Boles E, et al. (2004) Switching the mode of metabolism in the yeast *Saccharomyces cerevisiae. EMBO reports* 5: 532-537. doi: 10.1038/sj.embor.7400132.
[39] Cordier H, Mendes F, Vasconcelos I, Francois JM (2007) A metabolic and genomic study of engineered *Saccharomyces cerevisiae* strains for high glycerol production. *Metabolic Engineering* 9: 364-378.
[40] Daran-Lapujade P, Rossell S, van Gulik WM, Luttik MAH, de Groot MJL, et al. (2007) The fluxes through glycolytic enzymes in *Saccharomyces cerevisiae* are predominantly regulated at posttranscriptional levels. *Proceedings of the National Academy of Sciences* 104: 15753-15758 doi: 10.1073/pnas.0707476104.
[41] Daran-Lapujade P, Jansen MLA, Daran JM, van Gulik W, de Winde JH, et al. (2004) Role of Transcriptional Regulation in Controlling Fluxes in Central Carbon Metabolism of *Saccharomyces cerevisiae*. 279: 9125-9138.
[42] Basso TO, Dario MG, Tonso A, Stambuk BU, Gombert AK (2010) Insufficient uracil supply in fully aerobic chemostat cultures of *Saccharomyces cerevisiae* leads to respiro-fermentative metabolism and double nutrient-limitation. *Biotechnology Letter* 32: 973-977.
[43] Sanchez RG, Hahn-Hägerdal B, Gorwa-Grauslund MF (2010) PGM2 over-expression improves anaerobic galactose fermentation in *Saccharomyces cerevisiae. Microbial Cell Factories* 9: 40
[44] Ng CY, Jung MY, Lee JW, Oh MK (2012) Production of 2,3-butanediol in *Saccharomyces cerevisiae* by *in silico* aided metabolic engineering. *Microbial Cell Factories* 11:68.
[45] Marc J, Feria-Gervasio D, Mouret JR, Guillouet SE (2013) Impact of oleic acid as co-substrate of glucose on "short" and "long-term" Crabtree effect in *Saccharomyces cerevisiae Microbial Cell Factories* 12: 83.
[46] Kuyper M, Winkler AA, van Dijken JP, Pronk JT (2004) Minimal metabolic engineering of *Saccharomyces cerevisiae* for efficient anaerobic xylose fermentation: a proof of principle. *FEMS Yeast Research* 4: 655-664.
[47] Christen S, Sauer U (2011) Intracellular characterization of aerobic glucose metabolism in seven yeast species by ^{13}C £ux analysis and metabolomics. *FEMS Yeast Research* 11: 263-272.
[48] Schuurmans JM, L. Rossell SL, van Tuijl A, Bakker BM, Hellingwerf KJ, de Mattos MJT (2008) Effect of hxk2 deletion and HAP4 overexpression on fermentative capacity in *Saccharomyces cerevisiae. FEMS Yeast Research* 8: 95-203.
[49] Kong QX,Gu JG, Cao LM, Zhang AL, Chen X, Zhao XM (2006) Improved production of ethanol by deleting FPS1 and over-expressing GLT1 in *Saccharomyces cerevisiae. Biotechnology Letter* 28:2033–2038 doi: 10.1007/s10529-006-9185-5.
[50] Zou J, Guo XW, Shen T, Dong J, Zhang CY, et al. (2013) Construction of lactose-consuming *Saccharomyces cerevisiae* for lactose fermentation into ethanol fuel. *Journal of Industrial Microbiology and Biotechnology* 40:353-363.
[51] Suga HY, Matsuda F, Hasunuma T, Ishii J, Kondo A (2013) Implementation of a transhydrogenase-like shunt to counter redox imbalance during xylose fermentation in *Saccharomyces cerevisiae. Applied Microbiology Biotechnology* 97: 1669-1678. doi: 10.1007/s00253-012-4298-3.
[52] Madhavan A, Tamalampudi S, Srivastava A, Fukuda H, Bisaria VS, et al. (2009) Alcoholic fermentation of xylose and mixed sugars using recombinant *Saccharomyces cerevisiae* engineered for xylose utilization. *Applied Microbiology Biotechnology* 82: 1037-1047 doi: 10.1007/s00253-008-1818-2.

[53] Kong QX, Zhang AL, Cao LM, Chen X (2007) Over-expressing GLT1 in a gpd2Δ mutant of *Saccharomyces cerevisiae* to improve ethanol production. *Applied Microbiology Biotechnology* 75: 1361-1366 doi:10.1007/s00253-007-0948-2.
[54] Kong QX, Cao LM, Zhang AL, Chen X (2007) Over-expressing GLT1 in gpd1Δ mutant to improve the production of ethanol of *Saccharomyces cerevisiae. Applied Microbiology Biotechnology* 73: 1382-1386 doi: 10.1007/s00253-006-0610-4.
[55] Runquist D, Hahn-Hägerdal B, Bettiga M (2010) Increased ethanol productivity in xylose-utilizing *Saccharomyces cerevisiae* via a randomly mutagenized xylose reductase *Applied and Environmental Microbiology* 76: 7796-7802.
[56] van Maris AJA, Geertman JMA, Vermeulen A, Groothuizen MK, Winkler AA, Piper MDW, van Dijken JP, Pronk JT (2004) Directed evolution of pyruvate decarboxylase-negative *Saccharomyces cerevisiae*, yielding a C_2-Independent, glucose-tolerant, and pyruvate-hyperproducing yeast. *Applied and Environmental Microbiology* 70: 159-166.
[57] Kuyper M, Hartog MMP, Toirkens MJ, Almering MJH, Winkler AA, et al. (2005) Metabolic engineering of a xylose-isomerase-expressing *Saccharomyces cerevisiae* strain for rapid anaerobic xylose fermentation. *FEMS Yeast Research* 5: 399-409.
[58] Senac T, Hahn-Hägerdal B (1990) Intermediary Metabolite concentrations in xylulose- and glucose-fermenting *Saccharomyces cerevisiae* cells. *Applied Environmental Microbiology* 56: 120-126
[59] Postma E, Verduyn C, Scheffers WA, Van Dijken JP (1989) Enzymic analysis of the crabtree effect in glucose-limited chemostat cultures of *Saccharomyces cerevisiae. Applied Environmental Microbiology* 55: 468-477.
[60] Novy et al. : Process intensification through microbial strain evolution: mixed glucose-xylose fermentation in wheat straw hydrolyzates by three generations of recombinant *Saccharomy cescerevisiae. Biotechnology for Biofuels* 2014 7: 49.
[61] Demeke MM, Dietz H, Li YY, Foulquié-Moreno MR, Mutturi S, et al. (2013) Development of a D-xylose fermenting and inhibitor tolerant industrial *Saccharomyces cerevisiae* strain with high performance in lignocellulose hydrolysates using metabolic and evolutionary engineering. *Biotechnology for Biofuels* 6: 89.
[62] Koppram R, Albers E, Olsson L (2012) Evolutionary engineering strategies to enhance tolerance of xylose utilizing recombinant yeast to inhibitors derived from spruce biomass. *Biotechnology for Biofuels* 5: 32.
[63] Sanchez RG, Karhumaa K, Fonseca C, Nogué VS, Almeida JRM, et al. (2010) Improved xylose and arabinose utilization by an industrial recombinant *Saccharomyces cerevisiae* strain using evolutionary engineering. *Biotechnology for Biofuels* 3: 13.
[64] Matsushika A, Goshima T, Hoshino T (2014) Transcription analysis of recombinant industrial and laboratory Saccharomyces cerevisiae strains reveals the molecular basis for fermentation of glucose and xylose. *Microbial Cell Factories* 13: 16.
[65] Elbing K, Larsson C, Bill RM, Albers E, Snoep JL, et al. (2004) Role of hexose transport in control of glycolytic flux in *Saccharomyces cerevisiae. Applied and Environmental Microbiology* 70: 5323-5330.
[66] Gombert AK, dos Santos MM, Christensen B, Nielsen J (2001) Network Identification and Flux Quantification in the Central Metabolism of *Saccharomyces cerevisiae* under Different Conditions of Glucose Repression. *Journal of Bacteriology* 183:1441-1451 doi: 10.1128/JB.183.4.1441-1451.
[67] Rozpędowska E, Hellborg L, Ishchuk OP, Orhan F, Galafassi S, et al. (2010) Parallel evolution of the make-accumulate-consume strategy in *Saccharomyces* and *Dekkera* yeasts. *Nature Communication* 2: 302 /doi: 10.1038/ncomms1305.
[68] Bergdahl B, Gorwa-Grauslund MF, van Niel EWJ (2014) Physiological effects of over-expressing compartment-specific components of the protein folding machinery in xylose-fermenting *Saccharomyces cerevisiae. BMC Biotechnology* 14: 28 doi:10.1186/1472-6750-14-28.

[69] Peng BY, Shen Y, Li XY, Chen X, Hou J, et al. (2012) Improvement of xylose fermentation in respiratory-deficient xylose-fermenting *Saccharomyces cerevisiae. Metabolic Engineering* 14: 9-18.
[70] Simon Ostergaard S, WallØe KO, Gomes CSG, Olsson L, Nielsen J (2001) The impact of GAL6, GAL80, and MIG1 on glucose control of the GAL system in *Saccharomyces cerevisiae. FEMS Yeast Research* 1: 47-55.
[71] Alfenore S, Cameleyre X, Benbadis L, Bideaux C, Uribelarrea JL, Goma G, Molina-Jouve C, Guillouet SE (2004) Aeration strategy: a need for very high ethanol performance in *Saccharomyces cerevisiae* fed-batch process. *Applied Microbiology Biotechnology* 63:537-542 doi: 10.1007/s00253-003-1393-5.
[72] Zhang L, Tang Y, Guo ZP, Ding ZY, Shi GY (2011) Improving the ethanol yield by reducing glycerol formation using cofactor regulation in *Saccharomyces cerevisiae. Biotechnology Letter* 33:1375-1380 doi: 10.1007/s10529-011-0588-6.
[73] Wiebe MG, Rintala E, Tamminen A, Simolin H, Salusjärvi L, et al. (2008) Central carbon metabolism of *Saccharomyces cerevisiae* in anaerobic, oxygen-limited and fully aerobic steady-state conditions and following a shift to anaerobic conditions. *FEMS Yeast Research* 8: 140-154.
[74] Kim B, Du J, Eriksen DT, Zhao HM (2013) Combinatorial Design of a Highly Efficient Xylose-Utilizing Pathway in *Saccharomyces cerevisiae* for the Production of Cellulosic Biofuels. *Applied and Environmental Microbiology* 79: 931-941.
[75] Geertman JMA, van Maris AJA, van Dijken JP, Pronk JT (2006) Physiological and genetic engineering of cytosolic redox metabolism in *Saccharomyces cerevisiae* for improved glycerol production. *Metabolic Engineering* 8: 532-542.
[76] Boer VM, Tai SL, Vuralhan Z, Arifin Y, Walsh MC, et al. (2007) Transcriptional responses of *Saccharomyces cerevisiae* to preferred and non preferred nitrogen sources in glucose-limited chemostat cultures. *FEMS Yeast Research* 7:604-620 doi:10.1111/j.1567-1364.2007.00220.x.
[77] Agren R, Otero JM, Nielsen J (2013) Genome-scale modeling enables metabolic engineering of *Saccharomyces cerevisiae* for succinic acid production. *Journal of Industrial Microbiology and Biotechnology* 40: 735-747. doi: 10.1007/s10295-013-1269-3
[78] Chumnanpuen P, Nookaew I, Nielsen J (2013) Integrated analysis, transcriptome-lipidome, reveals the effects of INO- level (INO2 and INO4) on lipid metabolism in yeast. *BMC Systems Biology* 7(Suppl 3): S7.
[79] Kocharin K, Nielsen J (2013) Specific growth rate and substrate dependent polyhydroxybutyrate production in *Saccharomyces cerevisiae. AMB Express* 3: 18.
[80] Kocharin K, Siewers V, Nielsen J (2013) Improved polyhydroxybutyrate production by *Saccharomyces cerevisiae* through the use of the phosphoketolase pathway Biotechnology and Bioengineering 110: 2216-2224.
[81] Liu Z, Hou J, Martínez JL, Petranovic D, Nielsen J (2013) Correlation of cell growth and heterologous protein production by *Saccharomyces cerevisiae. Applied Microbiology Biotechnology* 97: 8955-8962. doi:10.1007/s00253-013-4715-2
[82] Chen Y, Daviet L,Schalk M, Siewers V, Nielsen J (2013) Establishing a platform cell factory through engineering of yeast acetyl-CoA metabolism. *Metabolic Engineering* 15: 48-54.
[83] Scalcinati G, Partow S, Siewers V, Schalk M, Daviet L, et al. (2012) Combined metabolic engineering of precursor and co-factor supply to increase α-santalene production by *Saccharomyces cerevisiae. Microbial Cell Factories* 11: 117.
[84] Kuyper M, Toirkens MJ, Diderich JA, Winkler AA, van Dijken JP, et al. (2005) Evolutionary engineering of mixed-sugar utilization by a xylose-fermenting *Saccharomyces cerevisiae* strain. *FEMS Yeast Research* 5: 925-934.
[85] Abbott DA, Knijnenburg TA, de Poorter LMI, Reinders MJT, Pronk JT, et al. (2007) Generic and specific transcriptional responses to different weak organic acids in anaerobic chemostat cultures of *Saccharomyces cerevisiae. FEMS Yeast Research* 7: 819-833 doi:10.1111/j.1567-1364.2007.00242.x.

[86] dos Santos MM, Thygesen G, KÖtter P, Olsson L, Nielsen J (2003) Aerobic physiology of redox-engineered *Saccharomyces cerevisiae* strains modified in the ammonium assimilation for increased NADPH availability. *FEMS Yeast Research* 4: 59-68.
[87] Bro C, Regenberg B, Lagniel G, Labarre J, Montero-Lomelí M, et al. (2003) Transcriptional, proteomic, and metabolic responses to Lithium in galactose-grown yeast cells. *The Journal of Biological Chemistr*y 278: 32141-32149.
[88] Roca C, Nielsen J, Olsson L (2003) Metabolic engineering of ammonium assimilation in xylose-fermenting *Saccharomyces cerevisiae* improves ethanol production. *Applied and Environmental Microbiology* 69: 4732-4736.
[89] Smits HP, Hauf J, MuÈller S, Hobley TJ, Zimmermann FK, et al. (2000) Simultaneous over-expression of enzymes of the lower part of glycolysis can enhance the fermentative capacity of *Saccharomyces cerevisiae. Yeast* 16: 1325-1334.
[90] Schuurmans JM, Boorsma A, Lascaris R, Hellingwerf KJ, de Mattos MJT (2008) Physiological and transcriptional characterization of *Saccharomyces cerevisiae* strains with modified expression of catabolic regulators. *FEMS Yeast Research* 8: 26-34.
[91] Schulze U, Lidén G, Nielsen J, Villadsen J (1996) Physiological effects of nitrogen starvation in an anaerobic batch culture of *Saccharomyces cerevisiae. Microbiology* 142: 2299-2310. doi:10.1099/13500872-142-8-2299.
[92] Costenoble R, Picotti P, Reiter L, Stallmach R, Heinemann M, et al. (2011) Comprehensive quantitative analysis of central carbon and amino-acid metabolism in *Saccharomyces cerevisiae* under multiple conditions by targeted proteomics. *Molecular Systems Biology* 7: 464 doi:10.1038/msb.2010.122.
[93] Klein CJL, Rasmussen JJ, Rønnow B, Olsson L, Jens Nielsen J (1999) Investigation of the impact of MIG1 and MIG2 on the physiology of *Saccharomyces cerevisiae. Journal of Biotechnology* 68: 197-212.
[94] Hector RE, Qureshi N, Hughes SR, Cotta MA (2008) Expression of a heterologous xylose transporter in a *Saccharomyces cerevisiae* strain engineered to utilize xylose improves aerobic xylose consumption. *Applied Microbiology Biotechnology* 80: 675-684.
[95] Nogué VS, Narayanan VI, Gorwa-Grauslund MF (2013) Short-term adaptation improves the fermentation performance of *Saccharomyces cerevisiae* in the presence of acetic acid at low pH. *Applied Microbiology Biotechnology* 97: 7517-7525. doi: 10.1007/s00253-013-5093-5
[96] Verduyn C, Postama E, Scheffers WA, van Dijke JP (1990) Physiology of *Saccharomyces cerevisiae* in anaerobic glucose-limited chemostat cultures. Journal of General Microbiology 136: 395-403.
[97] Pagliardini J, Hubmann G, Alfenore S, Nevoigt E, Bideauxand C, et al. (2013) The metabolic costs of improving ethanol yield by reducing glycerol formation capacity under anaerobic conditions in *Saccharomyces cerevisiae. Microbial Cell Factories* 12: 29.
[98] Johansson B, Hahn-Hägerdall B (2002) The non-oxidative pentose phosphate pathway controls the fermentation rate of xylulose but not of xylose in *Saccharomyces cerevisiae* TMB3001. *FEMS Yeast Research* 2: 277-282.
[99] Kümmel A, Ewald JC, Fendt SM, Jol SJ, Picotti P, Aebersold R, Sauer U, Zamboni N, Heinemann M (2010) Differential glucose repression in common yeast strains in response to HXK2 deletion. *FEMS Yeast Research* 10: 322-332.
[100] Albertin W, Marullo P, Aigle M, Dillmann C, de Vienne D, et al. (2011) Population size drives industrial *Saccharomyces cerevisiae* alcoholic fermentation and is under genetic control. *Applied and Environmental Microbiology* 77: 2772-784.
[102] Aboka FO, Heijnen JJ, van Winden WA (2009) Dynamic ^{13}C-tracer study of storage carbohydrate pools in aerobic glucose-limited *Saccharomyces cerevisiae* confirms a rapid steady-state turnover and fast mobilization during a modest stepup in the glucose uptake rate. *FEMS Yeast Research* 9: 191-201.

[103] Mensonides FIC, Schurrmans JM, de Mattos MJT, Hellingwerf KJ, Brul S (2002) The metabolic response of *Saccharomyces cerevisiae* to continuous heat stress. *Molecular Biology Reports* 29: 103-106.
[104] Sanchez RG, Hahn-Hägerdal B, Gorwa-Grauslund MF (2010) Cross-reactions between engineered xylose and galactose pathways in recombinant *Saccharomyces cerevisiae. Biotechnology for Biofuels* 3: 19.
[105] Weusthuis RA, Pronk JT, van den Broek PJ, van Dijken JP (1994) Chemostat cultivation as a tool for studies on sugar transport in yeasts. *Microbiology Review* 58: 616-630.
[106] Bergdahl B, Sandström AG, Borgströ m C, Boonyawan T, van Niel EWJ, et al. (2013) Engineering yeast hexokinase 2 for improved tolerance toward xylose-induced inactivation. *PLOS ONE* 8(9): e75055. doi:10.1371/journal.pone.0075055.
[107] Mapelli V, Hillestrøm PR, Patil K, Larsen EH, Olsson L (2012) The interplay between sulphur and selenium metabolism influences the intracellular redox balance in *Saccharomyces cerevisiae. FEMS Yeast Research* 1220–32.
[108] Feng X, Zhao H (2013) Investigating xylose metabolism in recombinant *Saccharomyces cerevisiae* via ^{13}C metabolic flux analysis. *Microbial Cell Factories* 12: 114.
[109] Almeida JRM, Bertilsson M, Hahn-Hägerdal B, Lidén G, Gorwa-Grauslund MF (2009) Carbon fluxes of xylose-consuming *Saccharomyces cerevisiae* strains are affected differently by NADH and NADPH usage in HMF reduction. *Applied Microbiology Biotechnology* 84:751-761 doi: 10.1007/s00253-009-2053-1.
[110] Spégel CF, Heiskanen AR, Kostesha N, Johanson TH, Gorwa-Grauslund MF, et al. (2007) Amperometric response from the glycolytic versus the pentose phosphate pathway in *Saccharomyces cerevisiae* Cells. *Analytical Chemistry* 79: 8919-8926.
[111] Almeida JRM, Röder A, Modig T, Laadan B, Lidén G, et al. (2008) NADH- vs NADPH-coupled reduction of 5-hydroxymethyl furfural (HMF) and its implications on product distribution in *Saccharomyces cerevisiae. Applied Microbiology Biotechnology* 78: 939-945.
[112] Karhumaa K, Fromanger R,Hahn-Hägerdal B, Gorwa-Grauslund MF (2007) High activity of xylose reductase and xylitol dehydrogenase improves xylose fermentation by recombinant *Saccharomyces cerevisiae. Applied Microbiology Biotechnology* 73:1039-1046. doi: 10.1007/s00253-006-0575-3.
[113] Jeppsson M, Bengtsson O, Franke K, Lee H, Hahn-Hägerdal B, et al. (2005) The expression of a Pichia stipitis Xylose reductase mutant with higher K_m for NADPH increases ethanol production from xylose in recombinant *Saccharomyces cerevisiae. Biotechnology and Bioengineering* 93: 665-673.
[114] Karhumaa K, Wiedemann B, Hahn-Hägerdal B, Boles E, Gorwa-Grauslund MF (2006) Co-utilization of L-arabinose and D-xylose by laboratory and industrial *Saccharomyces cerevisiae* strains. *Microbial Cell Factories* 5:18 doi:10.1186/1475-2859-5-18.
[115] Jeppsson M, Johansson B, Hahn-Hägerdal B, Gorwa-Grauslund MF (2002) Reduced oxidative pentose phosphate pathway fux in recombinant xylose-utilizing *Saccharomyces cerevisiae* strains improves the ethanol yield from xylose. *Applied and Environmental Microbiology* 68: 1604-1609.
[116] Jeppsson M, Johansson B, Jensen PR, Hahn-Hägerdal B, Gorwa-Grauslund MF (2003) The level of glucose-6-phosphate dehydrogenase activity strongly influences xylose fermentation and inhibitor sensitivity in recombinant *Saccharomyces cerevisiae* strains. *Yeast* 20: 1263-1272.
[117] Jeppsson M, Träff K, Johansson B, Hahn-Hägerdal B, Gorwa-Grauslund MF (2003) Effect of enhanced xylose reductase activity on xylose consumption and product distribution in xylose-fermenting recombinant *Saccharomyces cerevisiae. FEMS Yeast Research* 3:167-175.
[118] Träff-Bjerre KL, Jeppsson M, Hahn-Hägerdal B, Gorwa-Grauslund MF (2004) Endogenous NADPH-dependent aldose reductase activity influences product formation during xylose consumption i n recombinant *Saccharomyces cerevisiae. Yeast* 21: 141-150.

[119] Sonderegger M, Jeppsson M, Larsson C, Gorwa-Grauslund MF, Boles E, et al. (2004) Fermentation performance of engineered and evolved xylose-fermenting *Saccharomyces cerevisiae strains. Biotechnology and Bioengineering* 87: 90-98.
[120] Albers E, Larsson C, Lidén G, Niklasson C, Gustafsson L (1996) Influence of the Nitrogen Source on *Saccharomyces cerevisiae* anaerobic growth and product formation. *Applied and Environmental Microbiology* 62: 3187-3195.
[121] Scalcinati G, Otero JM, Van Vleet JRH, Jeffries TW, Olsson L, et al. (2012) Evolutionary engineering of *Saccharomyces cerevisiae* for efficient aerobic xylose consumption. *FEMS Yeast Research* 12: 582-597.
[122] Yang KM, Lee NR, Woo JM, Choi W, Zimmermann M, et al. (2012) Ethanol reduces mitochondrial membrane integrity and thereby impacts carbon metabolism of *Saccharomyces cerevisiae. FEMS Yeast Research* 12: 675-684.
[123] Klimacek M, Krahulec S, Sauer U, Nidetzky B (2010) Limitations in Xylose-Fermenting *Saccharomyces cerevisiae*, made evident through comprehensive metabolite profiling and thermodynamic analysis. *Applied and Environmental Microbiology* 76: 7566-7574.
[124] Runquist D, Hahn-Hägerdal B, Bettiga M (2009) Increased expression of the oxidative pentose phosphate pathway and gluconeogenesis in anaerobically growing xylose-utilizing *Saccharomyces cerevisiae. Microbial Cell Factories* 8:49 doi: 10.1186/1475-2859-8-49.
[125] von Meyenburg HK (1969) Energetics of the budding cycle of *Saccharomyces cerevisiae* during glucose limited aerobic growth. *Archeal Microbiology* 66: 289–303.
[126] Mashego MR, Jansen MLA, Vinke JL, van Gulik WM, Heijnen JJ (2005) Changes in the metabolome of *Saccharomyces cerevisiae* associated with evolution in aerobic glucose-limited chemostats. *FEMS Yeast Research* 5: 419-430.
[127] Frick O, Wittmann C (2005) Characterization of the metabolic shift between oxidative and fermentative growth in *Saccharomyces cerevisiae* by comparative ^{13}C flux analysis. *Microbial Cell Factories* 4:30 doi:10.1186/1475-2859-4-30.
[128] Brochado AR, Matos C, Møller BL, Hansen J, Mortensen UH, et al. (2010) Improved vanillin production in baker's yeast through in silico design. *Microbial Cell Factories* 9:84.
[130] Compagno C, Brambilla L, Capitanio D, Boschi F, Ranzi FM, et al. (2001) Alterations of the glucose metabolism in a triose phosphate isomerase-negative *Saccharomyces cerevisiae* mutant. *Yeast* 18: 663-670.
[131] Hanscho M, David E. Ruckerbauer DE, Chauhan N, Hofbauer HF, et al. (2012) Nutritional requirements of the BY series of *Saccharomyces cerevisiae* strains for optimum growth. *FEMS Yeast Research* 12:796-808.
[132] Boer VM, Tai SL, Vuralhan Z, Arifin Y, Walsh MC, et al. (2007) Transcriptional responses of *Saccharomyces cerevisiae* to preferred and non preferred nitrogen sources in glucose-limited chemostat cultures. *FEMS Yeast Research* 7: 604-620.
[133] van Maris AJA, Luttik MAH, Winkler AA, van Dijken JP, Pronk JT (2003) Overproduction of threonine aldolase circumvents the biosynthetic role of pyruvate decarboxylase in glucose-limited chemostat cultures of *Saccharomyces cerevisiae. Applied and Environmental Microbiology* 69:2094-2099.
[134] van der Aar PC, Lopes TS, Klootwijk J, Groeneveld Ph, van Verseveld HW, et al. (1990) Consequences of phosphoglycerate kinase overproduction for the growth and physiology of *Saccharomyces cerevisiae. Applied Microbiology and Biotechnology* 32: 577-587
[135] Mwesigyeb PK, Barford JP (1996) Batch growth and transport kinetics of utilization of mixtures of sucrose and maltose by *Saccharomyces cerevisiae. Journal of Fermentation and Bioengineering* 82: 101-108.
[137] Simon Ostergaard S, Lisbeth Olsson L, Jens Nielsen J (2000) In vivo dynamics of galactose metabolism in *Saccharomyces cerevisiae*: metabolic fluxes and metabolite levels. *Biotechnology and Bioengineering* 73:412-425.

[138] Aguilera J, Petit T, de Winde JH, Pronk JT (2005) Physiological and genome-wide transcriptional responses of *Saccharomyces cerevisiae* to high carbon dioxide concentrations. *FEMS Yeast Research* 5: 579-593.
[139] Tai SL, Daran-Lapujade P, Luttik MAH, Walsh MC, Diderich JA, et al. (2007) Control of the glycolytic flux in *Saccharomyces cerevisiae* grown at low temperature. *The Journal of Biological Chemistry* 282: 10243-10251.
[140] Schuurmans JM, Boorsma A, Lascaris R, Hellingwerf KJ, de Mattos MJT (2008) Physiological and transcriptional characterization of *Saccharomyces cerevisiae* strains with modified expression of catabolic regulators. *FEMS Yeast Research* 8: 26-34.
[141] Gutiérrez-Lomelí M, Torres-Guzmán JC, González-Hernández GA, Cira-Chávez LA, Pelayo-Ortiz C, et al. (2008) Over-expression of ADH1 and HXT1 genes in the yeast *Saccharomyces cerevisiae* improves the fermentative efficiency during tequila elaboration. *Antonie van Leeuwenhoek* 93: 363-371.
[143] Wang K, Mao ZG, Zhang GM, Zhang JH, Zhang HJ, et al. (2012) Influence of nitrogen sources on ethanol fermentation in an integrated ethanol-methane fermentation system. *Bioresource Technology* 120: 206-211.
[144] Wang JJ, Jiang JC, Jazwinski SM (2010) Gene regulatory changes in yeast during life extension by nutrient limitation. *Experimental Gerontology* 45: 621-631.
[145] Boer VM, Daran JM, Almering MJH, de Winde JH, Pronk JT (2005) Contribution of the *Saccharomyces cerevisiae* transcriptional regulator Leu3p to physiology and gene expression in nitrogen- and carbon-limited chemostat cultures. *FEMS Yeast Research* 5: 885-897.
[146] de Kok S, Yilmaz D, Suir E, Pronk JT, Daran JM, et al. (2011) Increasing free-energy (ATP) conservation in maltose-grown *Saccharomyces cerevisiae* by expression of a heterologous maltose phosphorylase. *Metabolic Engineering* 13: 518-526.
[147] De Nicola R, Hazelwood LA, De Hulster EAF, Walsh MC, Knijnenburg TA, et al. (2007) Physiological and Transcriptional Responses of *Saccharomyces cerevisiae* to zinc limitation in chemostat cultures. *Applied and Environmental Microbiology* 73:7680-7692 doi:10.1128/AEM.01445-07.
[148] Canelas AB, Ras C, Pierick A, van Gulik WM, Heijnen JJ (2011) An in vivo data-driven framework for classification and quantification of enzyme kinetics and determination of apparent thermodynamic data. *Metabolic Engineering* 13: 294-306.
[149] Boer VM, de Winde JH, Pronk JT, Piper MDW (2003) The Genome-wide Transcriptional Responses of *Saccharomyces cerevisiae* grown on glucose in aerobic chemostat cultures limited for Carbon, Nitrogen, Phosphorus, or Sulfur. *The Journal of Biological Chemistry* 278: 3265-3274.
[150] van Maris AJA, Bakker BM, Brandt M, Boorsma A, de Mattos MJT, et al. (2001) Modulating the distribution of £uxes among respiration and fermentation by overexpression of HAP4 in *Saccharomyces cerevisiae. FEMS Yeast Research* 1:139-149.
[151] Agrimi G, Mena MC, Izumi K, Pisano I, Germinario L, et al. (2013) Improved sake metabolic profile during fermentation due to increased mitochondrial pyruvate dissimilation. *FEMS Yeast Research* 14:249-260.
[152] Petschacher B, Nidetzky B (2008) Altering the coenzyme preference of xylose reductase to favor utilization of NADH enhances ethanol yield from xylose in a metabolically engineered strain of *Saccharomyces cerevisiae. Microbial Cell Factories* 7:9 doi: 10.1186/1475-2859-7-9.
[153] Vázquez-Lima F, Silva P, Barreiro A, Martínez-Moreno R, Morales P, et al. (2014) Use of chemostat cultures mimicking different phases of wine fermentations as a tool for quantitative physiological analysis. *Microbial Cell Factories* 13: 85.
[154] van den BJ, Akeroyd MR, van der Hoeven R, Pronk JT, de Winde JH, et al. (2009) Energetic limits to metabolic flexibility: responses of *Saccharomyces cerevisiae* to glucose-galactose transitions. *Microbiology* 155: 1340-1350.

[155] Antoniukas L, Grammel H, Sasnauskas K, Reichl U (2008) Profiling of external metabolites during production of hantavirus nucleocapsid protein with recombinant *Saccharomyces cerevisiae. Biotechnology Letter* 30: 415-420 doi: 10.1007/s10529-007-9577-1.
[157] Berovic M, Herga M (2007) Heat shock on *Saccharomyces cerevisiae* inoculums increases glycerol production in wine fermentation. *Biotechnology Letter* 29: 891-894 doi: 10.1007/s10529-007-9337-2.
[158] Albers E, Gustafsson L, Niklasson C, Lidén G (1998) Distribution of C-14-labelled carbon from glucose and glutamate during anaerobic growth of *Saccharomyces cerevisiae. Microbiology* 144: 1683-1690 doi: 10.1099/ 00221287-144-6-1683.
[159] Meinander N, Zacchi G, Hahn-Hägerdal B (1996) A heterologous reductase affects the redox balance of recombinant *Saccharomyce scerevisiae. Microbiology* 142: 165-172.
[160] Aguilera J, Petit T, de Winde JH, Pronk JT (2005) Physiological and genome-wide transcriptional responses of *Saccharomyces cerevisiae* to high carbon dioxide concentrations. *FEMS Yeast Research* 5: 579-593.
