# Supplementary material for: Physiologically Shrinking the Solution Space of a Saccharomyces cerevisiae Genome-Scale Model Suggests the Role of the Metabolic Network in Shaping Gene Expression Noise
Source: PLoS One. 2015 Oct 8;10(10):e0139590. doi: 10.1371/journal.pone.0139590 (PMC4598104; doi:10.1371/journal.pone.0139590)
Supplement: S3 File — Regression analysis of growth rate against glucose uptake rate in each segment (Figure A).Correlations between metabolic flux and gene expression noise (Figure B).Relationship between metabolic flux constraint (FSC) and gene expression noise (DM) (Figure C).The newly defined bounds (Table A).Regression analysis of growth rate versus glucose uptake rate in each segments. (y = αx +β; y is for growth rate and x is for glucose uptake rate(Table B). (PDF) [file pone.0139590.s004.pdf]

S3 File Supplementary Figures and Tables

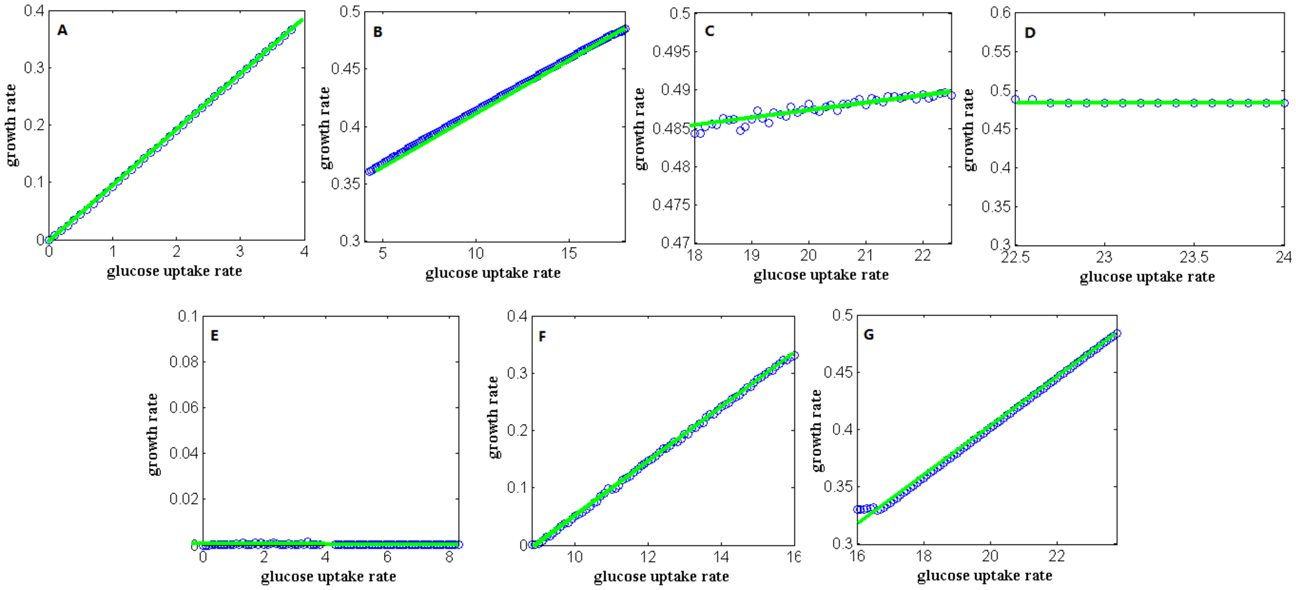

Figure A. Regression analysis of growth rate against glucose uptake rate in each segment. (A), (B), (C), (D) for the upper bound; (E), (F), (G) for the lower bound.

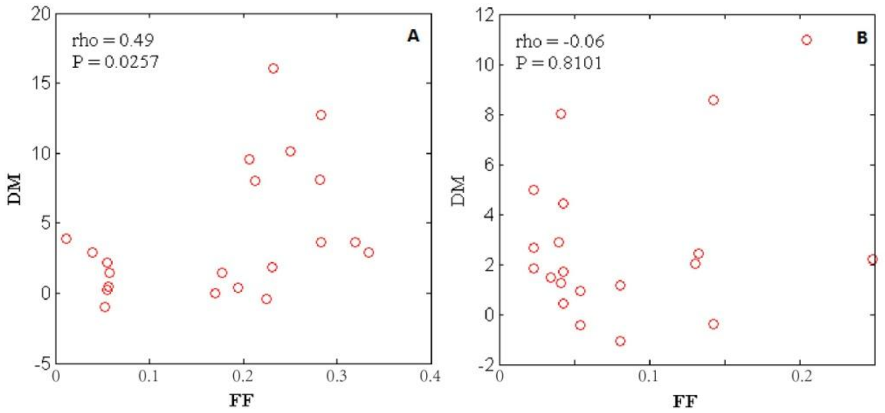

Figure B. Correlations between metabolic flux and gene expression noise. (A) is for the NER group and (B) is for ER group. The FF values were calculated by sampling a part of the shrunk space, where glucose uptake rate fluctuates within 18-20 mmol/(g (DW) ·h).

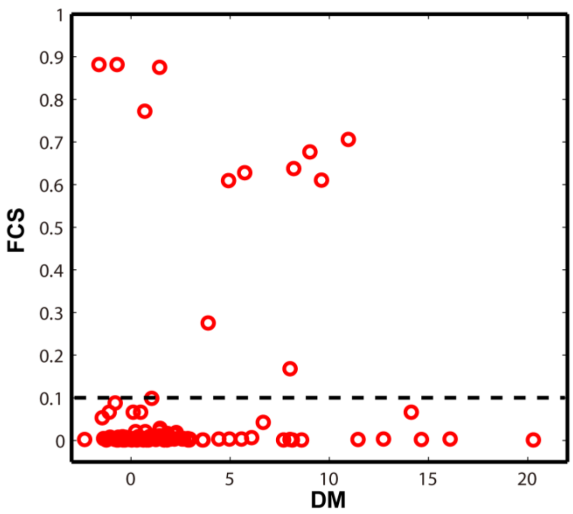

Figure C. Relationship between metabolic flux constraint (FSC) and gene expression noise (DM).

Table A. The newly defined bounds. O_2 consumption rate, ethanol production rate and glycerol secretion rate as a function of glucose uptake rate. (**y** = α **x** + β; **y** is for O_2 consumption rate, ethanol production rate and glycerol secretion rate; **x** is for glucose uptake rate)

		Upper bound		Lower bound	
		α	*β*	*α*	*β*
O2 consumption rate vs glucose uptake rate	< 4	**2.258**	**2**	**2.258**	**-1**
	> 4	**-0.473**	**10.79**	**-0.473**	**4.89**
ethanol production rate vs glucose uptake rate		**1.5824**	**1.5824**	**1.5824**	**-6.3296**
glycerol production rate vs glucose uptake rate		**0.25**	**0**	**0.02**	**0**

Table B. Regression analysis of growth rate versus glucose uptake rate in each segments. (**y** = α **x** + β; **y** is for growth rate and **x** is for glucose uptake rate)

bound type	glucose uptake rate	α	β
upper	0-4	**0.097**	**-0.001**
	4-18	**0.009**	**0.323**
	18-22.5	**0.001**	**0.465**
	22.5-24	**0**	**0.444**
lower	0-8	**0**	**0**
	8-16	**0.046**	**-0.415**
	16-24	**0.021**	**-0.019**
